# Supplementary figures and images for: Distinct stem-like cell populations facilitate functional regeneration of the Cladonema medusa tentacle
Source: PLoS Biol. 2023 Dec 21;21(12):e3002435. doi: 10.1371/journal.pbio.3002435 (PMC10734932; doi:10.1371/journal.pbio.3002435)

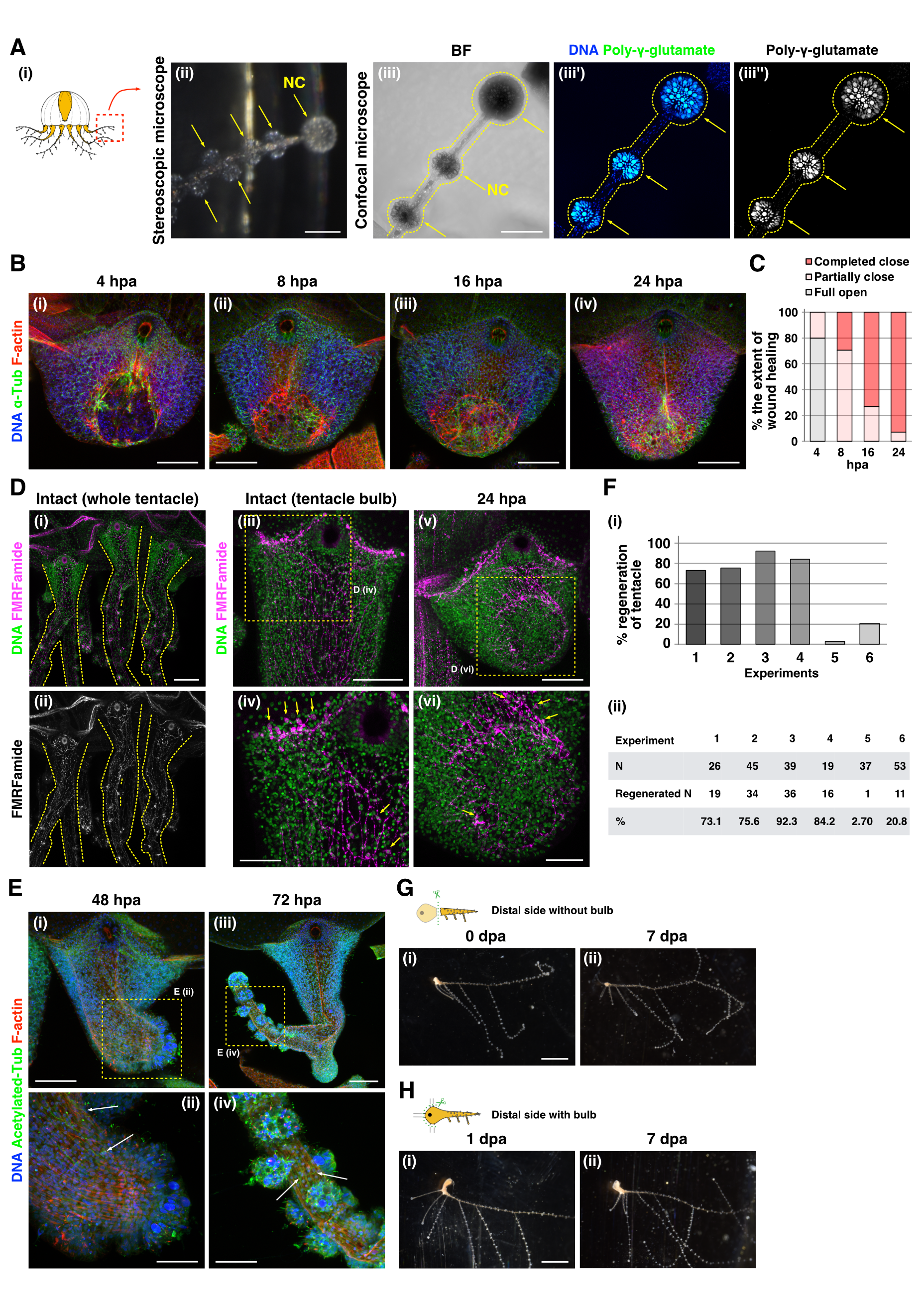

Supplement: S1 Fig — (A) Representative images of nematocyte clusters (NCs) in the medusa tentacle. Yellow arrows indicate nematocyte clusters that include mature nematocytes (Poly-γ-glutamate+). (B) The process of wound healing from 4 hpa to 24 hpa with Phalloidin and anti-α-Tubulin antibody. Phalloidin for F-actin (red) and α-Tubulin (green). (C) The extent of wound healing during tentacle regeneration. Full open: actin fibers not attached (Bi), partially close: disorganized actin fibers (Bii and Biii), completed close: actin fibers are fully attached (Biv); 4 hpa: n = 15 (tentacles), 8 hpa: n = 17, 16 hpa: n = 15, 24 hpa: n = 14. (D) Neural morphology in intact tentacle and the regenerating tentacle stained with the anti-FMRFamide antibody. Yellow allows indicate cell bodies of FMRFamide neurons. FMRFamide (magenta). (i, ii) Intact whole tentacle, (iii, iv) basal side of intact tentacle, (v, vi) regenerating tentacle at 24 hpa. (E) Neural morphology in the regenerating tentacle stained with the anti-acetylated-Tubulin antibody. White arrows indicate neural fibers; acetylated-Tubulin (green). (F) The rate of the tentacle regeneration after removing the bulb from canals. Difference of the regeneration rate between each experiment. (G) Images of the isolated tentacle without bulb at 1 dpa and 7 dpa. (H) Images of the isolated whole tentacle 1 dpa and 7 dpa. The numerical values that were used to generate the graphs in (C and F) can be found in S1 Data. Scale bars: (A, Di, Diii, Dv, Ei, and Eiii) 100 μm, (Div, Dvi, Eii, and Eiv) 50 μm, (G and H) 1 mm. (TIFF) [file pbio.3002435.s001.tiff]

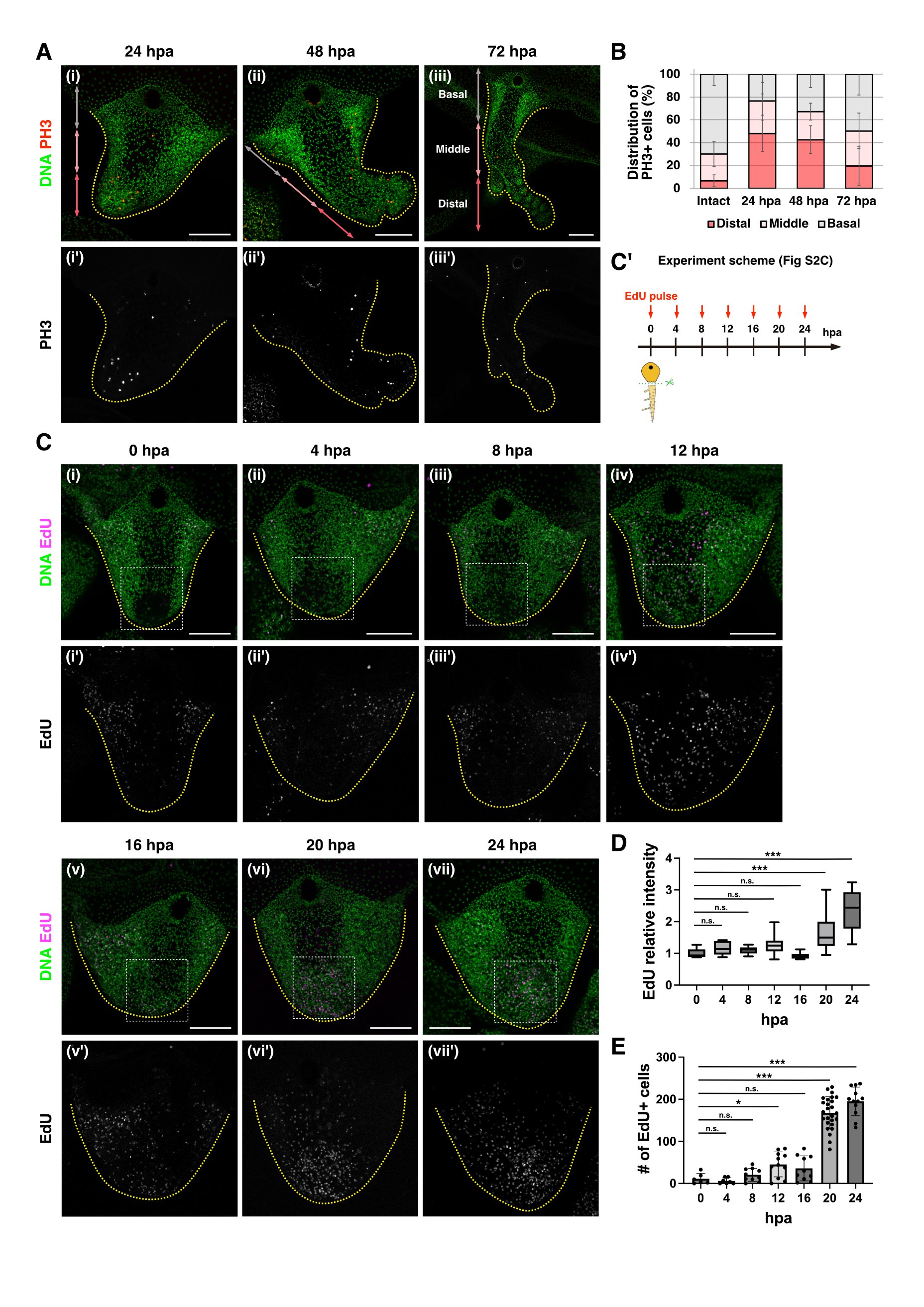

Supplement: S2 Fig — (A) Distribution of mitotic cells detected by anti-PH3 in regenerating tentacle. (B) Quantification of PH3+ cells’ distribution in regenerating tentacle based on the defined areas in (A). Intact: n = 11 (tentacles), 24 hpa: n = 10, 48 hpa: n = 9, 72 hpa: n = 8. (C) Distribution of S-phase cells in regenerating tentacle stained with EdU 1 h pulse labeling. EdU 1 h pulse labeling was performed at 0, 4, 8, 12, 16, 20, and 24 hpa as shown in the scheme. White dot squares show each quantification area (1502 μm2) in (D and E). (D) EdU relative intensity in blastema; 0 hpa: n = 6, 4 hpa: n = 7, 8 hpa: n = 10, 12 hpa: n = 11, 16 hpa: n = 10, 20 hpa: n = 26, 24 hpa: n = 12. (E) The number of EdU+ cells in blastema; 0 hpa: n = 6, 4 hpa: n = 7, 8 hpa: n = 10, 12 hpa: n = 11, 16 hpa: n = 10, 20 hpa: n = 26, 24 hpa: n = 12. *p < 0.05, ***p < 0.001. The numerical values that were used to generate the graphs in (B, D, and E) can be found in S1 Data. Scale bars: (A and C) 100 μm. (TIFF) [file pbio.3002435.s002.tiff]

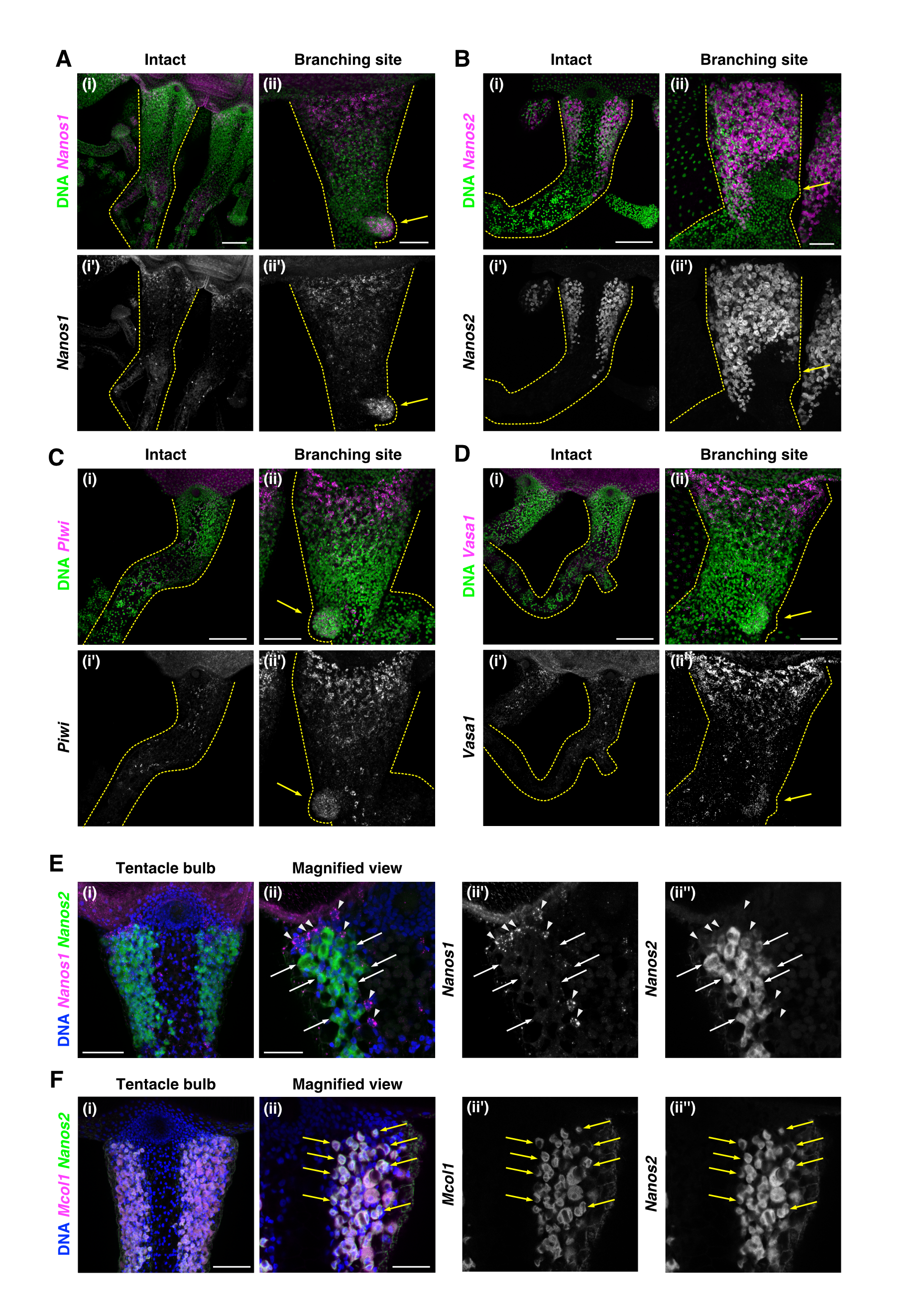

Supplement: S3 Fig — (A–D) Expression of Nanos1, Nanos2, Piwi, and Vasa1 in intact tentacles by FISH. Yellow arrows indicate the branching site. (E) Expression of Nanos1 and Nanos2 in intact tentacle by double FISH. White arrowheads indicate Nanos1+, and white arrows indicate Nanos2+. (F) Expression of Nanos2 and Mcol1 in intact tentacle by double FISH. Yellow arrows indicate representative co-expression of Nanos2 and Mcol1. Scale bars: (A–D) 100 μm, (Ei and Fi) 50 μm, (Eii and Fii) 25 μm. (TIFF) [file pbio.3002435.s003.tiff]

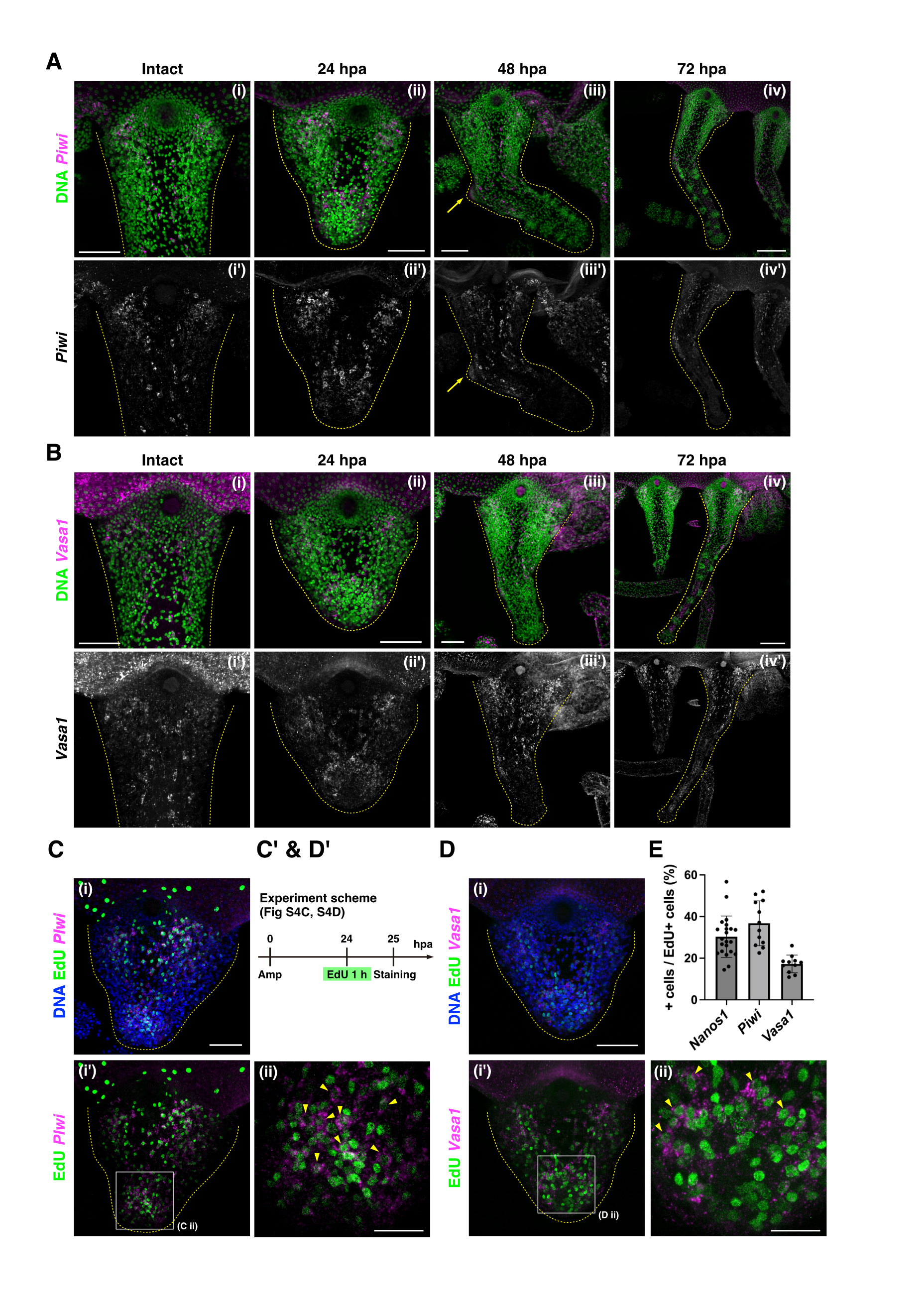

Supplement: S4 Fig — (A and B) Distribution of cells with stem marker genes (Piwi+ or Vasa1+) in intact tentacle and regenerating tentacle by FISH. Yellow arrow indicates the branching site. (C and D) Images of the regenerating tentacle co-labeled with Piwi or Vasa1 FISH and EdU 1 h pulse labeling at 24 hpa. EdU (green) and Piwi or Vasa1 (magenta). White squares show each quantification area (74.722 μm2) in (E). Yellow arrowheads indicate EdU+/Piwi+ or EdU+/Vasa1+ cell. (E) The rate of cells positive for stem cell marker genes in blastema. Nanos1: n = 22, Piwi: n = 12, Vasa1: n = 11. The data of “Nanos1” are the same as that in Fig 3F (Nanos1+/EdU+ cells in blastema). The numerical values that were used to generate the graphs in (E) can be found in S1 Data. Scale bars: (Aiv and Biv) 100 μm, (Ai-iii, Bi-iii, Ci, and Di) 50 μm, (Cii and Dii) 25 μm. (TIFF) [file pbio.3002435.s004.tiff]

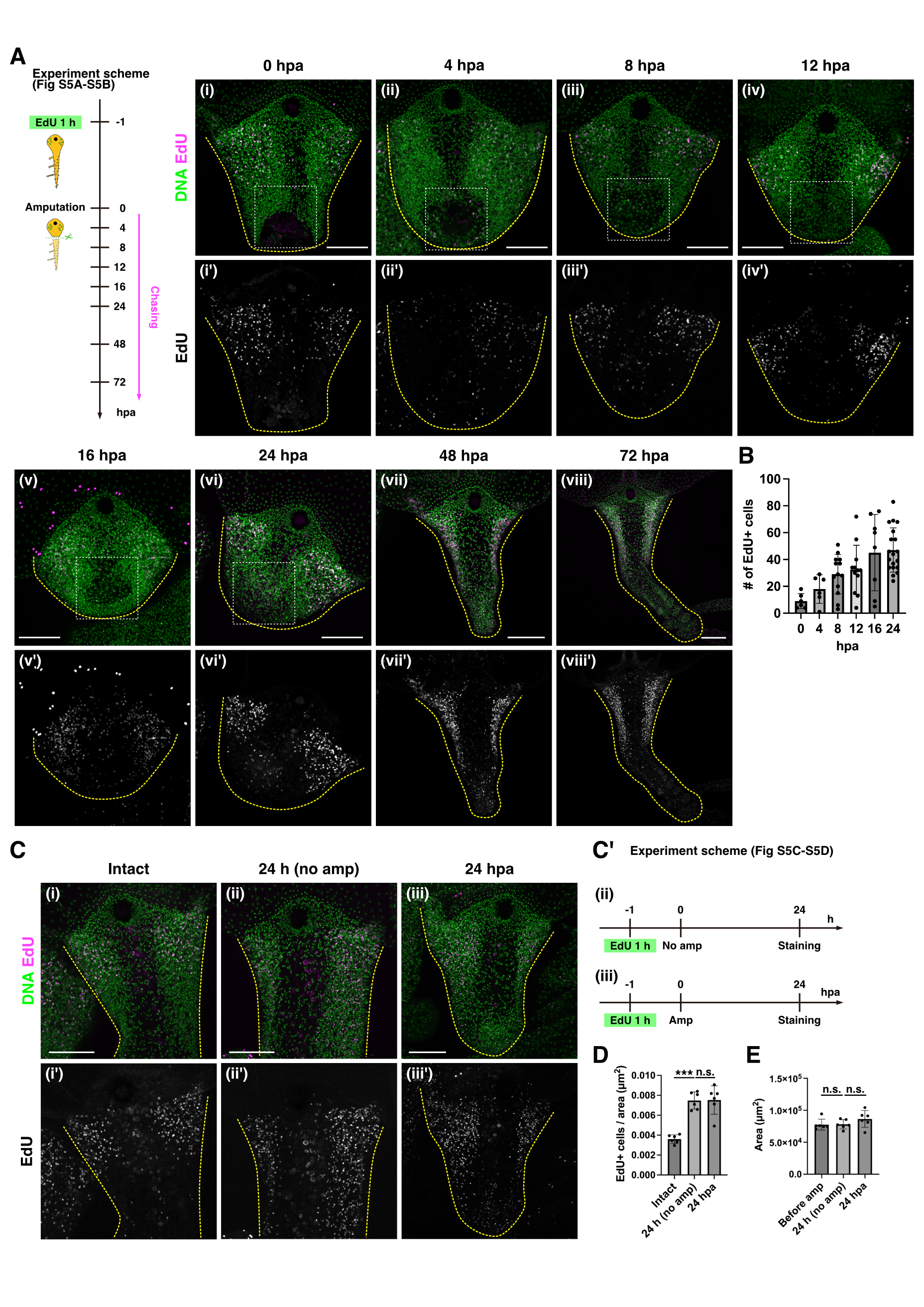

Supplement: S5 Fig — (A) Transition of labeled RHSCs during regeneration by chasing EdU+ cells. EdU 1 h labeling before amputation and chasing at 0, 4, 8, 12, 16, 24, 48, and 72 hpa. White dot squares show each quantification area (1502 μm2) in (B). (B) The number of EdU+ cells around blastema during regeneration; 0 hpa: n = 6, 4 hpa: n = 6, 8 hpa: n = 13, 12 hpa: n = 12, 16 hpa: n = 8, 24 hpa: n = 10. (C) The comparison of proliferative cell number in intact vs. no amputation vs. 24 hpa. EdU 1 h pulse labeling before amputation and chasing with amputation or without amputation (no amp). (D) The number of EdU+ cells labeled before amputation. Counted area is the whole regenerating tentacle at 24 hpa. Detailed information is in (E). Intact: n = 6 (tentacles), 24 h (no amp): n = 6, 24 hpa: n = 6. (E) Area size used for quantification. Note that the area of quantification at 24 hpa is similar. The numerical values that were used to generate the graphs in (B, D, and E) can be found in S1 Data. Unpaired two-tailed t test. ***p < 0.001. Scale bars: (A and C) 100 μm. (TIFF) [file pbio.3002435.s005.tiff]

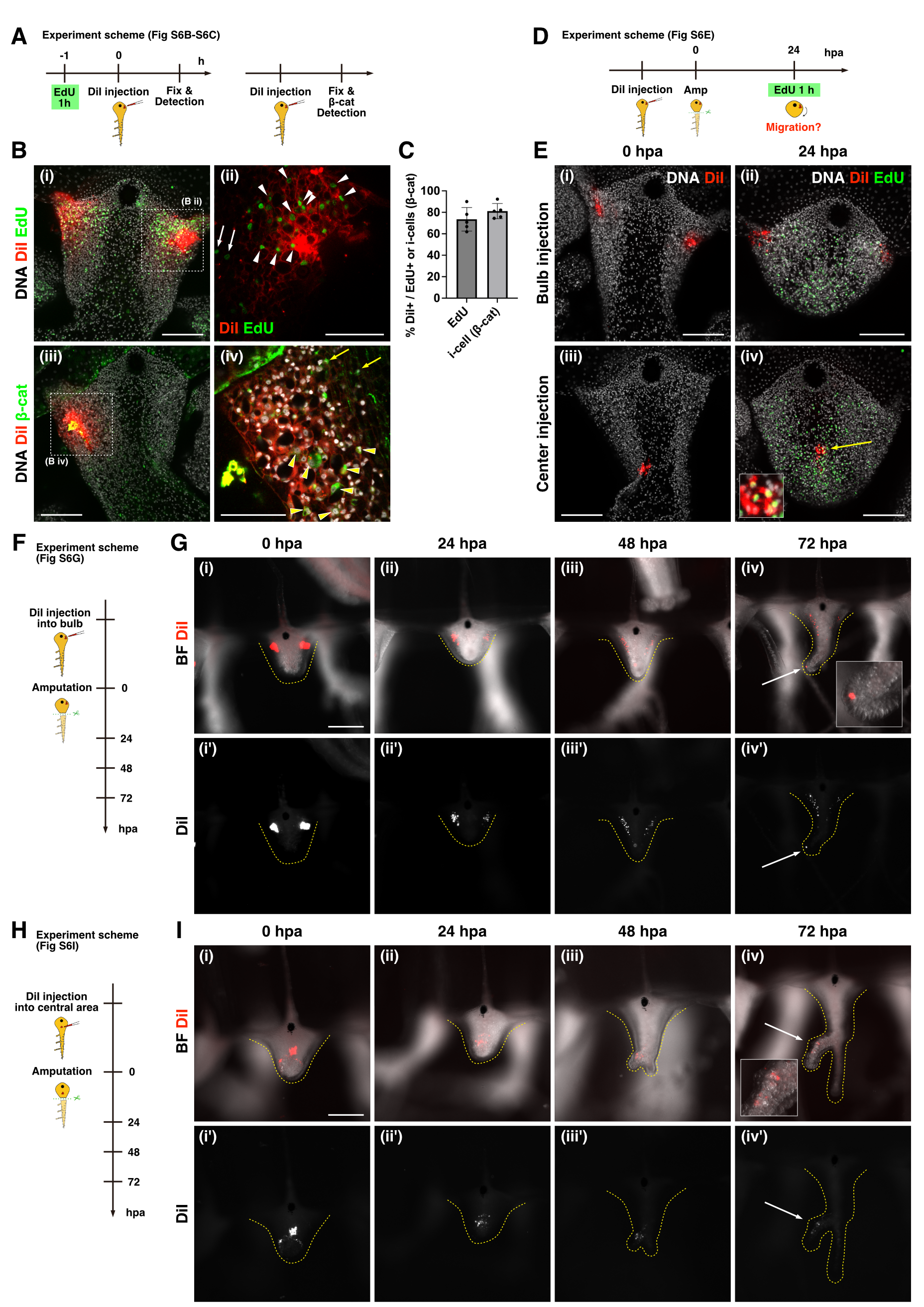

Supplement: S6 Fig — (A) Experimental scheme of DiI-labeled cell identification in the bulb with EdU or anti-β-catenin staining in (B). (B) Co-staining of DiI and EdU or anti-β-catenin in intact tentacle. White dot squares show each quantification area (74.722 μm2) in (C). White arrowheads indicate DiI+/EdU+ and white arrows indicate EdU+ only. Yellow arrowheads indicate DiI+/i-cells (β-catenin cytoplasmic signal+ cells) and yellow arrows indicate DiI-/i-cells. (C) Rate of DiI-labeled cells per proliferative cells or i-cells in bulb. Quantification of DiI+/EdU+ cells or DiI+/i-cells. DiI+/EdU+ cells: n = 5 (areas), DiI+/i-cells: n = 5. (D) Experimental scheme of chasing DiI-labeled cells in (E). EdU 1 h pulse labeling at 24 hpa. (E) (i and ii) Little migration of DiI-labeled cells in bulb from the moment of amputation to 24 hpa. n = 9/9. (iii and iv) DiI-labeled cells in center area that merged with EdU (yellow arrow). n = 10/12. (F) Experimental scheme for chasing DiI-labeled cells in the bulb in (G). (G) Distribution change of DiI-labeled cells in the bulb during tentacle regeneration of an animal. White arrow indicates migration of labeled cells to nematocyte cluster at distal side. n = 6/10. (H) Experimental scheme for chasing DiI-labeled cells in the central area of tentacle in (I). (I) Distribution change of DiI-labeled cells in central area during tentacle regeneration of an animal. White arrow indicates DiI-labeled cells in the newly regenerated tentacle. n = 17/19. The numerical values that were used to generate the graphs in (C) can be found in S1 Data. Scale bars: (G and I) 250 μm, (Bi, Biii, and E) 100 μm, (Bii and Biv) 50 μm. (TIFF) [file pbio.3002435.s006.tiff]

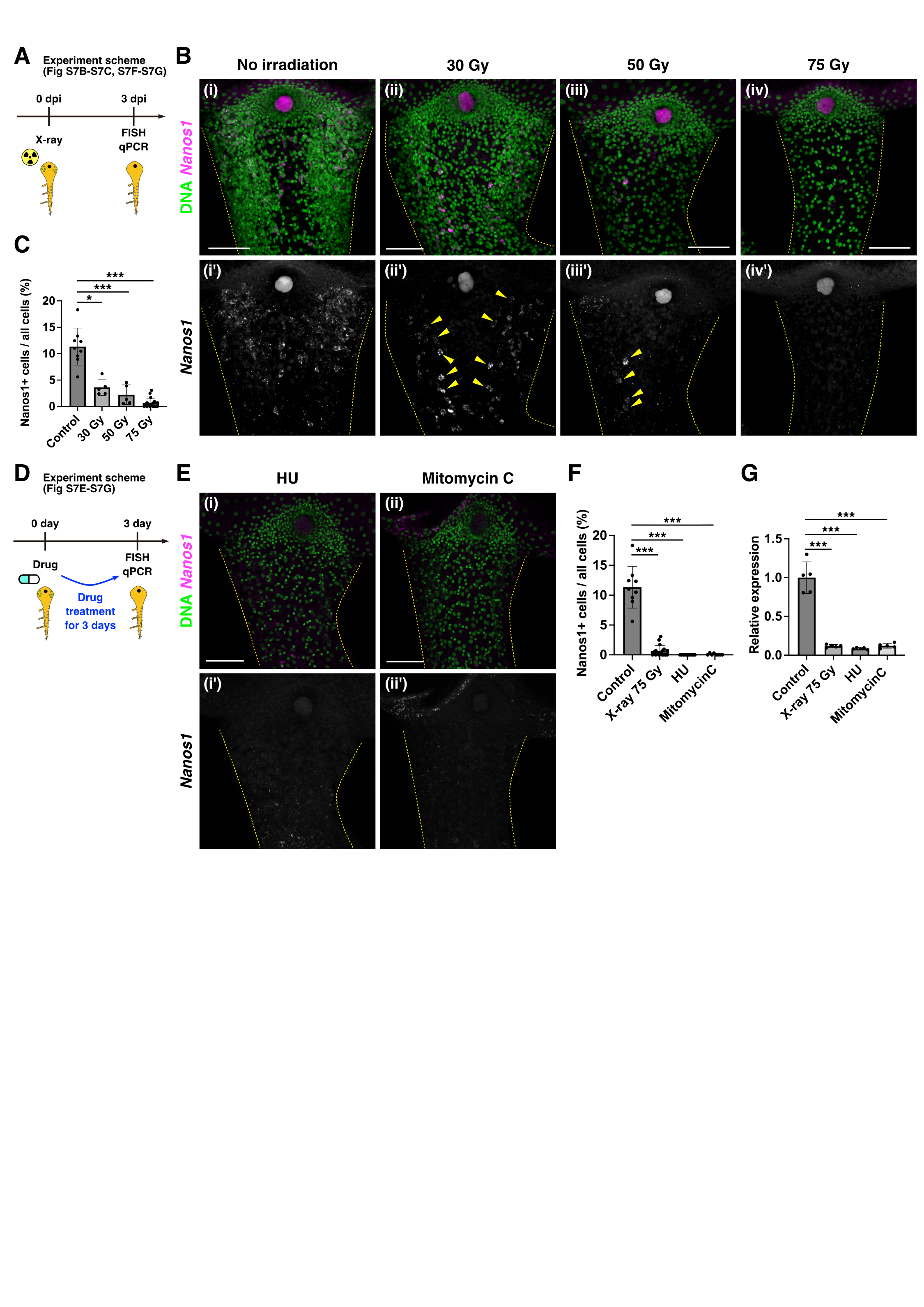

Supplement: S7 Fig — (A) Experimental scheme of X-ray irradiation (30, 50, and 75 Gy). FISH and qPCR at 3 days post-irradiation (3 dpi). (B) Expression of Nanos1 in intact tentacle at 3 dpi by FISH. (C) Quantification of Nanos1+ cells number in tentacle. Quantification area is the entire tentacle in confocal images. Control: n = 9, 30 Gy: n = 5, 50 Gy: n = 5, 75 Gy: n = 17. (D) Experimental scheme of drug treatments (HU 10 mM or Mitomycin C 30 μm). FISH and qPCR after 3 days of treatment. (E) Expression of Nanos1 in intact tentacle after 3 days drug treatments by FISH. (F) Quantification of Nanos1+ cells in tentacle. Quantification area is the entire tentacle in confocal images. Control: n = 9, 75 Gy: n = 17, HU: n = 10, Mitomycin C: n = 8. The data of “Control” and “X-ray 75 Gy” are the same as that in S7C Fig (Control and 75 Gy, respectively). (G) Relative expression of Nanos1 after irradiation and drug treatments by qPCR. The numerical values that were used to generate the graphs in (C, F, and G) can be found in S1 Data. Unpaired two-tailed t test. *p < 0.05, ***p < 0.001. Scale bars: (B and E) 50 μm. (TIFF) [file pbio.3002435.s007.tiff]

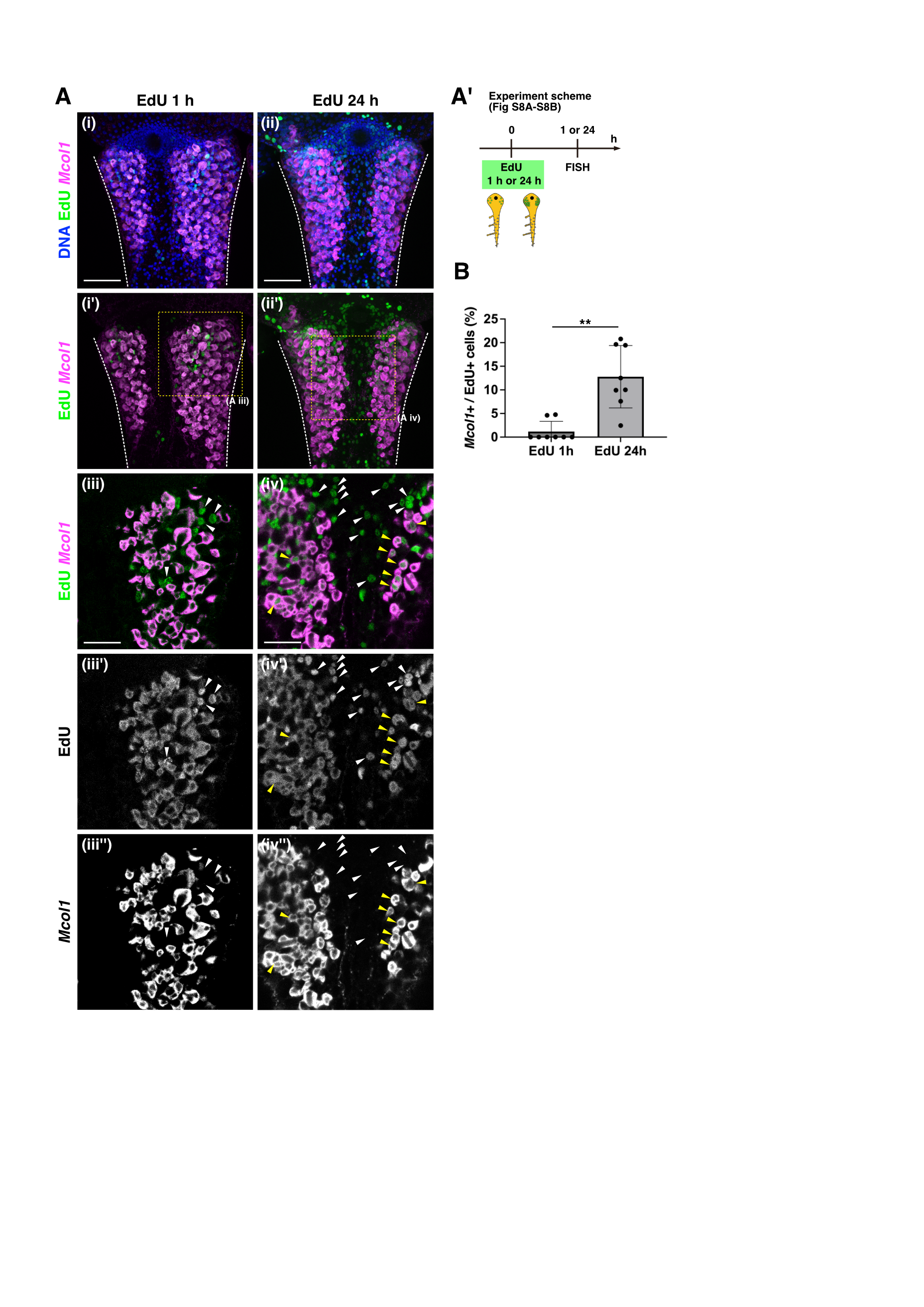

Supplement: S8 Fig — (A) Nematocyte progenitor cells in the bulb with dual staining of Mcol1 FISH and EdU staining. EdU 1 h or 24 h pulse labeling. The expression level of Mcol1 is extremely high such that the Mcol1 signal invades the wavelength of the EdU signal. White arrows indicate EdU+ cells and yellow arrowheads indicate Mcol1+/EdU+ cells. (B) The rate of Mcol1/EdU+ cells in intact tentacle. Quantification area is the entire tentacle in single section of confocal images. 1 h: n = 8 (tentacles), 24 h: n = 8. The numerical values that were used to generate the graphs in (B) can be found in S1 Data. Unpaired two-tailed t test. **p < 0.005. Scale bars: (A) 50 μm. (TIFF) [file pbio.3002435.s008.tiff]

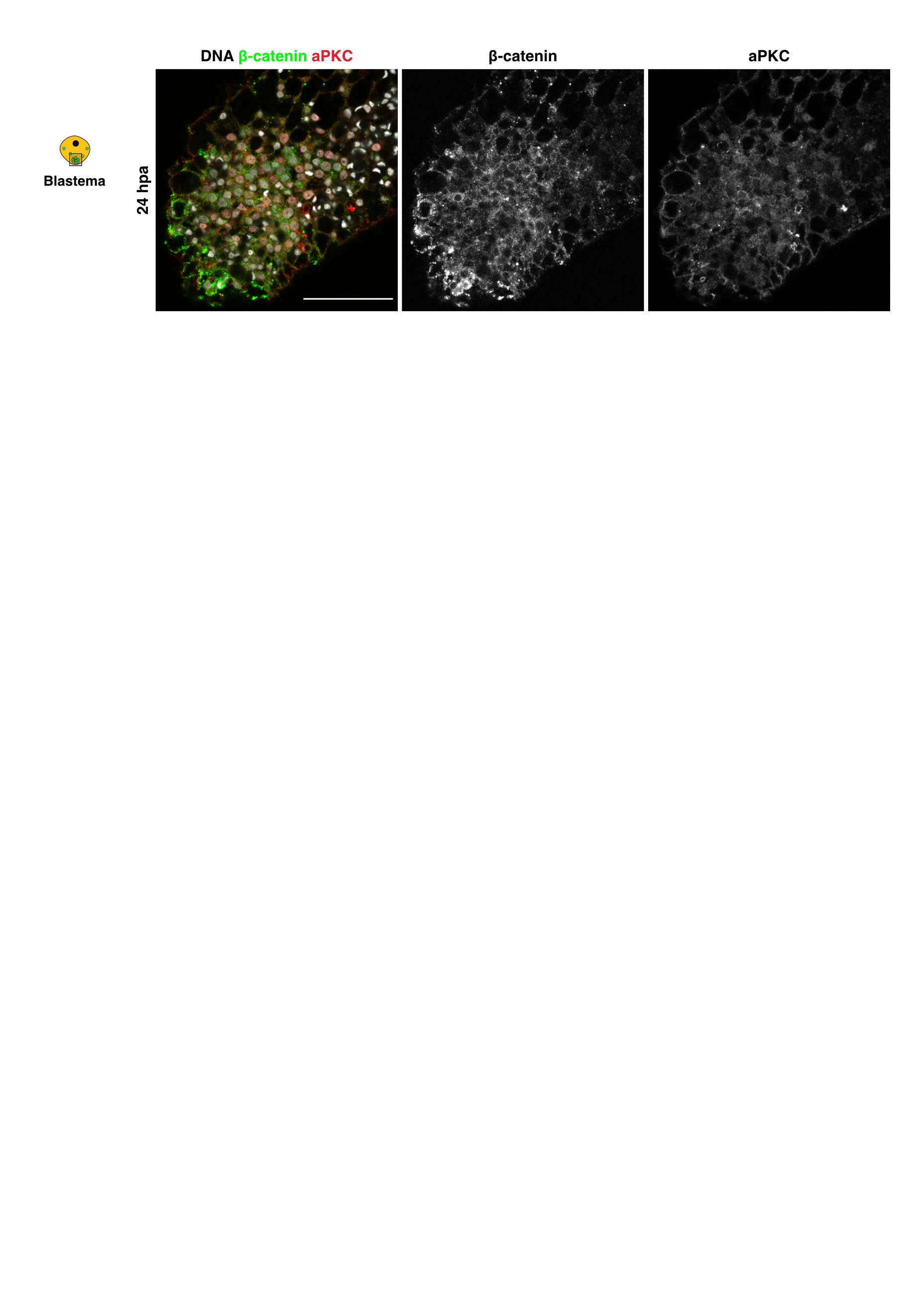

Supplement: S9 Fig — Identification of cell types in blastema stained by β-catenin and aPKC antibody staining. Scale bar: 50 μm. (TIFF) [file pbio.3002435.s009.tiff]

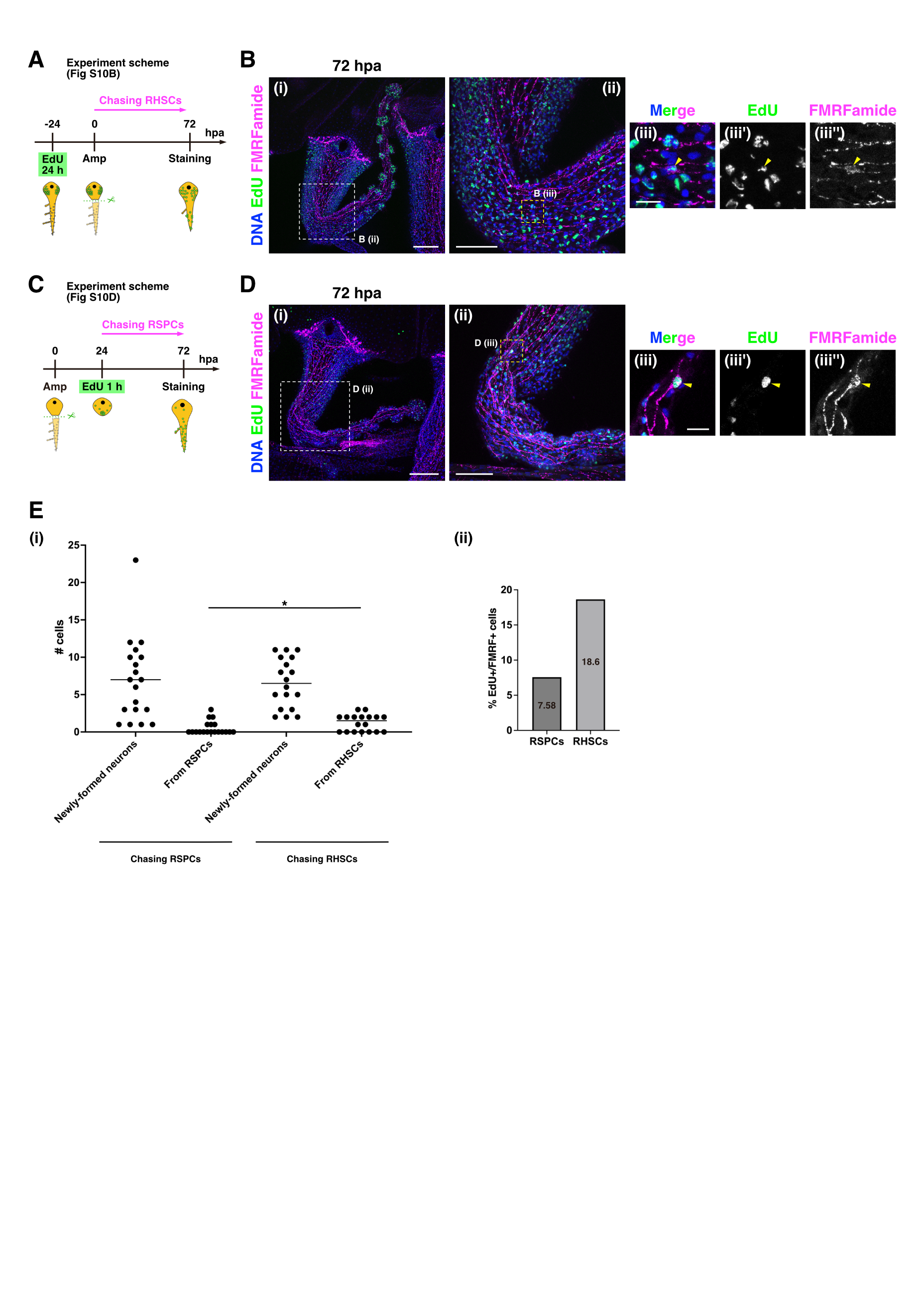

Supplement: S10 Fig — (A) Experimental scheme of chasing RHSCs in (B). (B) Neuron derived from RHSCs by co-staining with EdU and anti-FMRFamide antibody at 72 hpa. Yellow arrowhead indicates EdU+ and FMRFamide+ cell. (C) Experimental scheme of chasing RSPCs in (D). (D) Neuron derived from RSPCs by co-staining with EdU and anti-FMRFamide antibody at 72 hpa. Yellow arrowhead indicates EdU+ and FMRFamide+ cell. (E) (i) The number of neurons in each chasing experiment. Quantification area is the entire tentacle in confocal images (Bii and Dii). RSPCs: n = 19 (images), RHSCs: n = 18. (ii) Rate of EdU+/FMRFamide+ cell in (Ei). The numerical values that were used to generate the graphs in (E) can be found in S1 Data. Unpaired two-tailed t test. *p < 0.05. Scale bars: (Bi and Di) 100 μm, (Bii and Dii) 50 μm. (TIFF) [file pbio.3002435.s010.tiff]

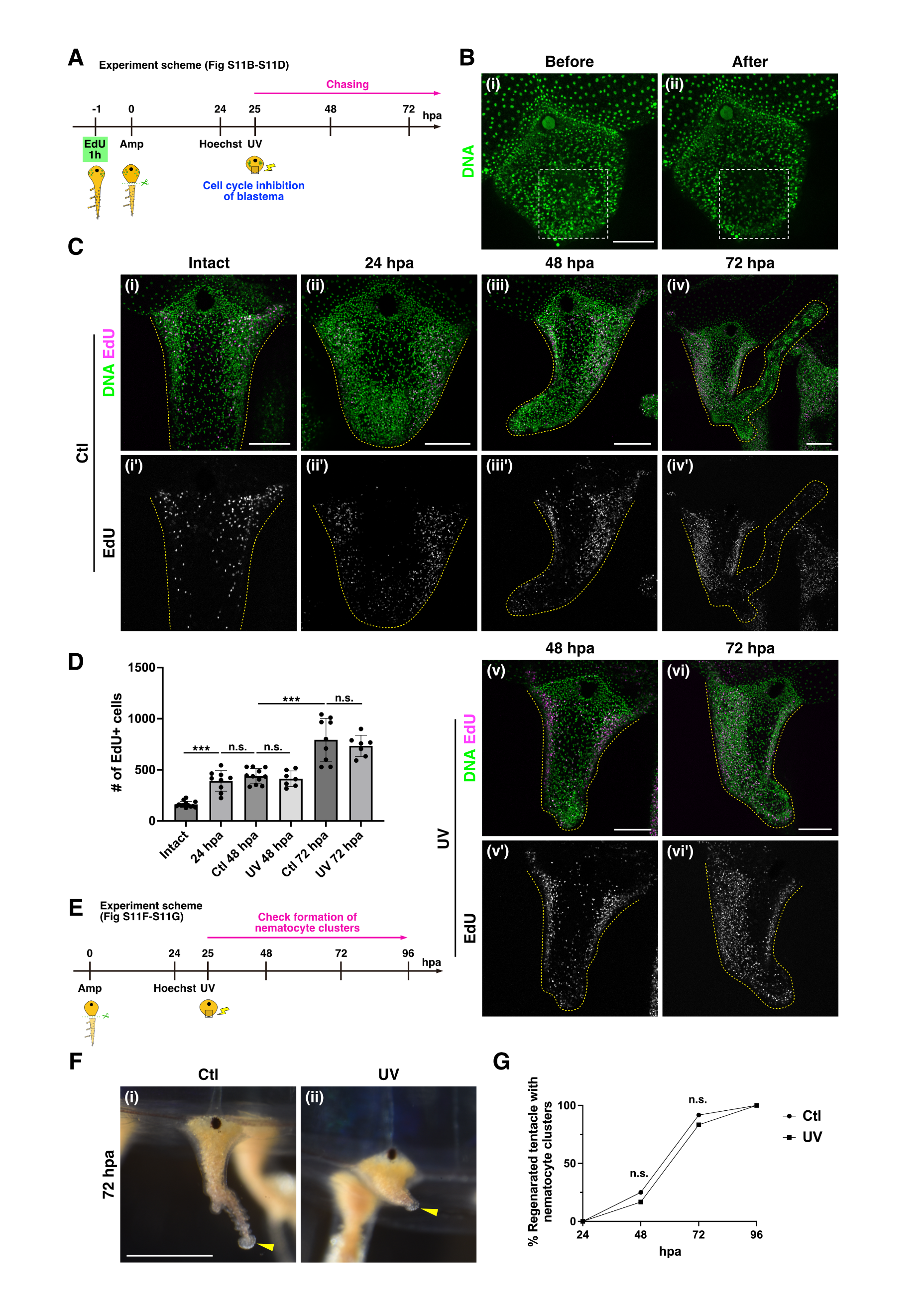

Supplement: S11 Fig — (A) Experimental scheme depicting the combination of EdU chasing and UV exposure in (B–D). (B) Bleaching of Hoechst signal only in UV exposure area at 24 hpa. White dot square is UV exposure area. (C) Distribution of EdU-labeled cells from intact to 72 hpa, Ctl (no UV) vs. UV. (D) The number of EdU+ cells during regeneration, Ctl (no UV) vs. UV. Intact: n = 13 (tentacles), 24 hpa: n = 9, 48 hpa Ctl: n = 11, 48 hpa UV: n = 7, 72 hpa Ctl: n = 9, 72 hpa UV: n = 7. (E) Experimental scheme depicting nematogenesis monitoring after UV exposure in (F and G). (F) Representative images of Ctl vs. UV at 72 hpa. Yellow arrowheads show nematocyte clusters. (G) Timing of nematocyte cluster formation during tentacle regeneration, Ctl vs. UV. Each tentacle: n = 12. The numerical values that were used to generate the graphs in (D and G) can be found in S1 Data. Unpaired two-tailed t test. ***p < 0.001. Scale bars: (F) 500 μm, (B and C) 100 μm. (TIFF) [file pbio.3002435.s011.tiff]
