# Supplementary material for: Distinct stem-like cell populations facilitate functional regeneration of the Cladonema medusa tentacle
Source: PLoS Biol. 2023 Dec 21;21(12):e3002435. doi: 10.1371/journal.pbio.3002435 (PMC10734932; doi:10.1371/journal.pbio.3002435)
Supplement: Attachment — Submitted filename: Response to reviewers-final3.pdf [file pbio.3002435.s013.pdf]

Response to reviewers' comments for the manuscript *PBIOLOGY-D-22-02819R1*

<u>Response to Reviewer #1</u>

Reviewer #1: Fujita et al. studied putative stem cells acting during tentacle regeneration in the hydrozoan jelly Cladonema pacificum. Despite the relatively small number of studied hydrozoans, it appears that their stem cell systems (collectively known as i-cells) have different properties in different genera. Therefore, studying i-cells in an additional hydrozoan is of particular interest.

The authors applied EdU/BrdU incorporation and immunofluorescence to label and track putative stem cells in regenerating tentacles. They also used X-ray irradiation to partially ablate cycling cells. They find that a blastema is established 24 hours post amputation (hpa), similar to other cnidarians. The authors provide a description of blastema cells. Using mRNA in situ hybridization, they show that Nanos and Piwi genes are expressed in the corresponding areas. Interestingly, they report that blastema cells fulfil only specific roles in regeneration, namely providing new epithelial cells but no nematocytes. Contrary to previous views, they conclude that blastema cells in Cladonema appear de novo at the injury site through dedifferentiation rather than being derived from resident tentacle bulb i-cells. Overall, the experiments are generally well performed, and the text mostly reads well. The description of Cladonema regeneration is an important resource for future work on this species. Specific comments are listed below.

***Comment 1.1.1**: The authors' conclusion that blastema cells appear de novo post amputation by dedifferentiation is not supported by their data for the following reasons.*

Response 1.1.1

We thank the reviewer for their generally positive remarks. While the reviewer claimed that we proposed dedifferentiation as a source of repair-specific proliferative cells, our conclusion was in fact of a different nature. Rather we clearly stated in the Discussion section that repair-specific proliferative cells are derived from "mitotic re-entry of quiescent/slow-cycling i-cells or dedifferentiation from differentiated cells into i-cells." Although we are not able to discriminate between these two possibilities at the current stage due to technical limitations using *Cladonema*, the novelty of our findings is that two different stem-like cell populations are involved in tentacle regeneration—specifically that repair-specific proliferating cells are distinct from resident homeostatic stem cells.

Overall, the reviewer seemed to prefer the migration scenario wherein resident stem cells contribute to blastema, but in the revised manuscript, we have bolstered the evidence to show that migration of resident stem cells is not sufficient to form blastema (Figures 4, S5, S6

and S7). Since the aim of our work is not to focus on the point that "*blastema cells appear de novo by dedifferentiation,*" in the following comments, we have mainly addressed two points: 1) most blastema cells are not derived from resident stem cells and 2) most resident stem cells do not migrate to form blastema.

Comment 1.1.2*: Intact animals were labeled using a 1-hour EdU incubation. Assuming that all i-cells in the tentacle bulb continuously cycle with a cell cycle duration of 24 hours (as in other hydrozoans), and that S-phase takes about a 1/3-1/2 of the cycle, a 1-hour incubation would label no more than 30-50% of all cycling i-cells (less if not all i-cells cycle that fast). The authors then amputated the tentacles and 24 hours later incubated the animals in BrdU for 1 hour that would, again, label only 30-50% of all cycling cells. Hence, if all blastema cells were descendants of resident tentacle bulb i-cells that migrated to the amputation site, one would expect about 16-25% of blastema cells to be double labeled 24 hpa. The authors report 12.6% double positive cells. These figures are consistent with the migration scenario rather than with dedifferentiation. If the latter were the case, one would expect none or only very few double labeled cells in the blastema.*

Response 1.1.2

This is a critical comment, but once again, we have to emphasize that we do not intend to conclude that dedifferentiation is the source of repair-specific proliferative cells.

To determine the contribution of resident stem cells to blastema formation, we have investigated both blastema formation and the migration of resident stem cells on a more granular time scale. EdU pulse experiments that label proliferating cells revealed that cell proliferation increased at the regenerating tip after 20 hpa when wound healing was completed, and became more active at 24 hpa (Revised Fig. S2C-S2E). We tracked the resident stem cells with EdU pulse labeling before amputation and found that, compared to the number of EdU^+ cells in blastema at 24 hpa (n=195.0 $\pm$ 33.9 Fig. S2E), the number of EdU^+ cells that migrate from resident stem cells to blastema (n=56.1 $\pm$ 17.8 Fig. S5B) is significantly smaller, suggesting that resident stem cells were insufficient for blastema formation. These results indicate that, although there is some contribution of resident stem cells in supplying proliferative cells that form blastema, most of the blastema cells are derived from other cellular sources.

It is also possible that the short 1-hour EdU incubation was not sufficient to label most resident stem cells in the EdU/BrdU double-chase experiment (Revised Fig. 4A-4D). We have thus performed an additional experiment in which we increased the duration of the EdU

incubation time from 1 hour to 24 hours and found that 13.6% of the BrdU$^+$ cells were EdU$^+$ resident stem cells in the 1 hour EdU incubation, while 26.9% of the BrdU$^+$ cells were EdU$^+$ cells in the 24 hour EdU incubation (Revised Fig. 4Cii, 4D, 5Bii, 5Biv, and 5C). This result shows that even under 24-hour conditions in which most resident stem cells must be labeled, more than 70% of the proliferative cells are distinct from resident stem cells. These results together strongly suggest that migration of resident stem cells into the bulb is not sufficient to form blastema. We have added the following sentences to the text in the revised manuscript.

> (Revised Manuscript; Page 18, Line 622-624)
> "Importantly, only 13.6% of BrdU$^+$ RSPCs were EdU$^+$ in blastema, supporting the notion that the migration of RHSCs may not be sufficient to form blastema (Fig 4Bi, 4Cii, and 4D)."

> (Revised Manuscript; Page 20, Line 681-687)
> "After EdU labeling for 24 h, followed by amputation, most EdU$^+$ cells remained in the bulb, and 26.9% of the BrdU$^+$ RSPCs were EdU$^+$ in the blastema, indicating that the majority of RSPCs are different lineage than RHSCs (Fig 5B ii, 5Biv, and 5C). This result is consistent with the results of the chasing experiment using short duration EdU labeling (Fig 4A-4D), further supporting the conclusion that the migration of RHSCs in the bulb is not sufficient to form blastema."

Comment 1.1.3*: Not all tentacle bulb S-phase cells are stem cells; many of them are transit amplifying progeny (e.g., nematoblasts) that are not expected to contribute to the cycling cells in the blastema (i.e., incorporate BrdU) 24 hpa because they would exit the cell cycle within 24 hours after EdU labeling. This would reduce the number of expected double labeled cells under the migration hypothesis, making the observed 12.6% inconsistent with the authors' dedifferentiation model.*

Response 1.1.3

It is true that the transit amplifying progeny including nematoblasts among EdU$^+$ cells would not be co-labeled with BrdU in blastema once they exit the cell cycle. However, nematoblasts must be considered if they migrate to the blastema region. As we have stated in **Response 1.1.2**, migration of resident stem cells is not a major determinant of blastema formation. Furthermore, in the revised manuscript, we have performed detailed cell tracking in the bulb, including resident stem cells by DiI injection, and shown that the labeled cells do not migrate to the

blastema from the time of amputation to 24 hpa (Revised Fig. S6A-S6G). Based on these results, we consider that the transit amplifying progeny like nematoblasts generally do not participate in blastema formation in addition to resident stem cells.

Comment 1.1.4*: Quiescent or slow cycling cells that re-enter the cell cycle post injury (the authors mention this possibility) would further reduce the numbers of double-labeled cells 24 hpa, making the dedifferentiation hypothesis even less likely.*

Response 1.1.4

As pointed out, repair-specific proliferative cells can arise not only from the dedifferentiation of neighboring differentiated cells but also from the cell cycle re-entry of quiescent/slow cycling cells. In fact, considering this possibility, we clearly described these two possibilities for the origin of repair-specific proliferative cells in the Discussion of our manuscript. However, as we mentioned in the Response 1.1.1, we cannot discriminate these two possibilities because the current available tools do not allow us to track a specific cell lineage in the *Cladonema* medusa and thus left as they are for future studies.

Comment 1.1.5*: Figure 2 i' and I'' show that S-phase in the blastema at 24 hpa comes at the expense of cycling cells in the tentacle bulb. This observation is consistent with a scenario in which many cycling tentacle bulb i-cells had migrated to the blastema. If dedifferentiation were the case, one would not expect tentacle bulb i-cells to reduce their proliferation rates.*

Response 1.1.5

In the original Figure 2A(ii) that showed the regenerating tentacle at 24 hpa, fewer EdU^+ cells were distributed in the bulb, and most EdU^+ cells accumulated in the amputated region, so it seems that the reviewer concluded that S-phase cells in the blastema comes at the expense of cycling cells in the tentacle bulb. However, this is likely due to the fact that we showed the EdU images of 24-72 hpa (Figure 2Aii-iv in the original manuscript), which were the results of 1 h incubation with EdU 20 μM, not the results of 1 h incubation with EdU 150 μM, which were used in other experiments. Another issue is that the signal of EdU in the tentacle bulb, which corresponds to resident stem cells, was not so high compared to blastema, so the brightness of these EdU signal was adjusted by ImageJ/Fiji for better visibility (Revised Fig. 2Ai). On the other hand, the signal of EdU was quite strong in blastema at 24 hpa, and we tried to avoid saturation of the signal intensity at blastema, which caused the signal in the bulb to become

darker. This may have given the impression that EdU+ cells in the bulb were moving to the injury site.

In the revised manuscript, we have replaced these images with the ones obtained by 1h incubation with EdU 150 μM (Revised Fig. 2A, 2B), which are consistent with other EdU pulse labeling experiments (Revised Fig. 2D, 2G-2I, 3E, 4A-4C, 4I, 5B, 5F, 6B, 6E, S2C, S4C-S4D, S5A-S5C, S6B, S6E, S8A, S10A-S10D, and S11C). As a result, EdU+ cells in the bulb were clearly detected at 24 hpa, although the signal of these EdU+ cells was weaker than that of blastema (Revised Fig. 2Aii). We have also quantified the relative intensity of EdU signal (Fig. R1) and found a strong signal as the evidence of highly proliferative population. Notably, the proliferation rate in resident stem cells in the bulb did not reduce and rather tended to increase after amputation of the tentacle (Revised Fig. 2D-2F), further supporting a scenario where resident stem cells do not migrate to blastema. Our new results together suggest that blastema contains highly proliferative cells, which are different from resident stem cells.

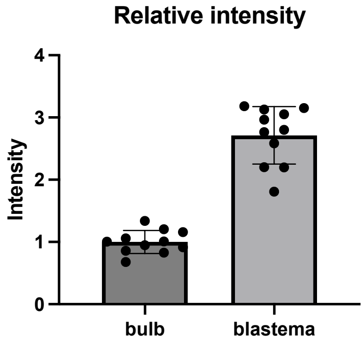

Fig. R1: Quantification of the relative intensity of EdU in blastema vs bulb area. EdU (150 μM/1 h) pulse labeling at 24 hpa. The quantification area is 75 μm^2.

Comment 1.1.6*: An additional argument against the dedifferentiation hypothesis is that 24 hours might be too short a time for somatic cells to dedifferentiate.*

Response 1.1.6

Dedifferentiation has been observed in various organ regeneration in different species, and the initiation time of dedifferentiation after injury is distinct among different species and organs [1]. For example, during zebrafish fin regeneration, dedifferentiation begins at about 12 hours after injury [2] while during zebrafish heart regeneration, loss of sarcomeres in muscles and cell cycle re-entry, both of which indicate a hallmark of cardiomyocyte dedifferentiation, are observed 7 days after injury [3,4]. In contrast, during the limb regeneration of adult newt, blastema begins to form 4-5 days after injury [5], and similarly, cell cycle re-entry in muscle is

also observed 4 days after injury [6]. Furthermore, the recent study on dedifferentiation in the cnidarian polyp *Hydractinia* has shown that it takes 3 days for cell cycle re-entry and 6 days for stem cell marker expression during head regeneration [7]. Given that the time required for dedifferentiation is diversified among species and even among organs in the same species, the possibility that dedifferentiation occurs within 24 hours during tentacle regeneration in *Cladonema* jellyfish cannot be denied.

Comment 1.1.7: *Irradiation that was used to eliminate resident i-cells wasn't effective enough as many i-cells remained (Figure S5C), making the results of these experiments inconclusive with respect to the source of i-cells in the blastema.*

Response 1.1.7

We thank the reviewer for pointing out this issue. In the original Figure S5C, after 30 Gy X-ray irradiation, *Nanos1*$^{+}$ stem cells still remained while the number of *Nanos1*$^{+}$ cells significantly decreased. In this experimental condition, it is possible that the remaining resident stem cells contribute to blastema formation. To avoid this possibility, we have explored conditions that can remove most resident stem cells and found that 75 Gy X-ray irradiation causes near-total loss of *Nanos1*$^{+}$ resident stem cells 3 days post-irradiation (dpi), which was confirmed by FISH and qPCR (Revised Fig. S7A-S7C and S7G). In addition to X-ray irradiation, we have further established a pharmacological treatment wherein *Nanos1*$^{+}$ resident stem cells were almost completely eliminated after incubation with hydroxyurea (HU) 10 mM for 3 days or Mitomycin C 30 μM for 3 days (Revised Fig. S7D-S7G). In order to test whether blastema can appear after the removal of resident stem cells, we examined whether proliferative cells still accumulate to the wound site in these experimental conditions. At 24 hpa, EdU^{+} proliferative cells accumulated to the regenerating tip, although the number of EdU^{+} cells decreased slightly. (Revised Fig. 4H-4J). These results indicate that blastema can form even in the absence of resident stem cells in the bulb and that most of the blastema cells are derived from non-resident stem cell lineage. We have revised the following sentence to explain the elimination of resident stem cells in the text:

> (Revised Manuscript; Page 19, Line 650-667)
> "We first determined a condition that would eliminate resident stem cells after exposure to different doses of X-ray irradiation (30, 50, 75 Gy) by monitoring expression of the stem cell marker *Nanos1*. FISH staining of *Nanos1* showed that *Nanos1*$^{+}$ cells decreased in an X-ray dose dependent manor, and most *Nanos1*$^{+}$ cells

were removed at 75 Gy at 3 days post-irradiation (dpi) (S7A-S7C Fig). qPCR analysis further confirmed significant reduction of *Nanos1* mRNA levels at 3 dpi (S7G Fig). We thus amputated the tentacle at 3 dpi and investigated the blastema formation by EdU pulse labeling (Fig 4H). At 24 hpa, blastema successfully formed regardless of X-ray irradiation, although the rate of EdU^+ cells in the blastema decreased slightly (Fig 4I and 4J). In order to support the conclusion derived from X-ray irradiation, we also performed pharmacological treatments using hydroxyurea (HU) or Mitomycin C, both of which are used to eliminate i-cells in other hydrozoan species [8–10]. The results of FISH and qPCR confirmed that most *Nanos1*$^+$ cells are removed by HU or Mitomycin C treatment for 3 days (Fig S7D-S7G). We then examined blastema formation after HU or Mitomycin C treatment and found that blastema formed at 24 hpa, consistent with the results of X-ray irradiation (Fig 4I and 4J)."

Comment 1.1.8*: Next, the authors used DiI labeling of tentacle bulb tissue and find no labeled cells in the blastema 24 hpa. They take this as evidence for dedifferentiation. However, for this to be true they would have to show that the dye effectively labels i-cells in the tentacle bulb. i-cells are 'hidden' in interstitial spaces, and it is possible that only epithelial cells were labeled by the authors' method. Furthermore, nematocyte progenitors that do migrate to the blastema from the tentacle bulb according to the authors should have been visible if all cell types were labeled by DiI. Finally, Figure S4C ii shows only few DiI-labeled cells and all (or nearly all) of them are EdU negative, inconsistent with i-cell labeling.*

Response 1.1.8

Firstly, as we have now repeatedly stated, we did not take the result of DiI labeling experiment as evidence of dedifferentiation but rather used it as a corroborating result to demonstrate that the contribution of resident stem cells to blastema formation is minimal. However, as the reviewer points out, in the original Figure S4C, we did not have a clear result from which to infer whether DiI labeling could stain resident stem cells (i-cells) in the bulb. We have thus performed both EdU labeling and β-catenin antibody staining in the same sample to confirm the cell-type of DiI+ cells in the bulb. We found that more than 75% of DiI labeled cells in the bulb are either EdU^+ cells or cells with i-cells morphology, as revealed by β-catenin cytoplasmic signal (Revised Fig. S6A-S6C). These results indicate that at least some portion of resident i-cells are labeled by DiI injection into the bulb.

To further confirm the origin of blastema cells, we injected DiI into either the bulb or the center of the tentacle to chase these DiI^+ cell populations while monitoring proliferating

cells in blastema by EdU labeling (Revised Fig. S6D, S6E). We found that DiI^+ cells did not merge with EdU^+ cells in blastema when injected into the bulb (Revised Fig. S6Ei and S6Eii), whereas DiI^+ cells that were labeled in the center of the tentacle merged with EdU^+ cells in blastema (Revised Fig. S6Eiii and S6Eiv). (Note that DiI^+ cells in the center of the tentacle merged with EdU^+ cells in more than 80% of the tentacles observed, n=10/12 {Revised Fig. S6D-S6E}). These results suggest that blastema cells are not derived from resident stem cells in the bulb, but rather from the cells located close to the injury site. In fact, when DiI was injected into the bulb, DiI^+ cells did not migrate to the blastema area of the regenerating tentacle, but rather DiI^+ cells migrated to the tip of the regenerating tentacle including the nematocyte cluster (Revised Fig. S6G), supporting the idea that resident stem cells in the bulb differentiate into nematocytes, not regenerating epithelial cells. In contrast, when injected into the central tentacle area, DiI^+ cells were initially distributed near the blastema region and then distributed to the regenerating apical side (Revised Figure S6I), suggesting that blastema cells preferentially differentiate into epithelial cells. We have added the following sentences to the text to explain the minimal contribution of resident stem cells based on DiI labeling experiments.

> (Revised Manuscript; Page 17-18, Line 599-609)
> "To confirm the cell-type of DiI^+ cells in the bulb, we first combined EdU staining or β-catenin antibody staining with DiI injection (S6A-S6C Fig) to show that more than 75% of EdU^+ cells or i-cells (β-catenin cytoplasm signal) were DiI^+ in the bulb (S6A-S6C Fig), indicating that the majority of resident i-cells are labeled by DiI injection. After a 24 h chase, DiI^+ cells were still localized near the bulb, not merged with EdU^+ cells in blastema (S6D and S6E Fig). In contrast, when DiI was injected into the central area near the amputation site, DiI^+ cells overlapped with EdU^+ cells, participating in blastema formation (S6D and S6E Fig). These results, taken together with the EdU chase results, suggest that resident stem cells do not migrate to the blastema region."

Comment 1.1.9*: Lastly, the different nature of blastema cells as compared to tentacle bulb i-cells is interesting; however, it does not necessarily support the dedifferentiation hypothesis. An alternative interpretation of these results is that blastema cells are mainly committed epithelial progenitors (derived from tentacle bulb i-cells) while tentacle bulb i-cells are stem cells with broad developmental potential, like in other hydrozoans.*

Response 1.1.9

As articulated above, we do not expect the different natures of blastema cells and resident stem cells to support the dedifferentiation scenario and rather have considered their differences to be evidence in support of distinct features of these stem-like populations. We confirmed that resident stem cells in the bulb differentiate into both epithelial cells and nematocytes, suggesting that they behave as multipotent stem cells, as in other hydrozoans (Revised Fig. 5A-5G). In contrast, repair-specific proliferative cells in blastema mainly contribute to newly generated tentacles by differentiating into epithelial cells (Revised Fig. 6A-6C and S6H-S6I). Although the detailed molecular characteristics of repair-specific proliferative cells remains unspecified, the majority of them exhibit clear i-cell morphology (Revised Fig. 3A-3B), suggesting that they are a part of hydrozoan i-cells based on current definition. Importantly, there is no clear marker for committed epithelial progenitors in cnidarians such as *Cladonema*, but instead these repair-specific proliferative cells heterogeneously express stem cell markers *Nanos1, Piwi,* and *Vasa1* (Revised Fig. 3E-3F and S4C-S4E). Intriguingly, we also found that repair-specific proliferative cells in blastema can differentiate into neurons in the regenerating tentacle (Revised Fig. S10C-S10D). We thus prefer to consider these proliferating blastema cells as lineage-restricted or bipotent stem cells based on i-cell morphological characteristics (Revised Fig. 3A-3B) as well as differentiation potency (Revised Fig. 6A-6C and S10C-S10D), unlike the multipotent resident stem cells in the bulb.

Comment 1.1.10*: The authors should revise their main conclusion by toning-down the corresponding statements on the source of blastema cells throughout the paper, including the title. It is of course possible that, in addition to migration, dedifferentiation also contributes to the blastema, but this remains a hypothesis, not supported by their current data.*

Response 1.1.10

Considering the data in the revised manuscript, especially the additional evidence obtained during revision, it is clear that resident stem cells in the bulb do not significantly contribute to blastema formation and that proliferative cells in blastema behave in a repair-specific manner and exhibit a nature distinct from resident stem cells. Our main conclusion is that two stem-like cell populations contribute to tentacle regeneration with different roles, and thus we respectfully disagree with the suggestion to tone down the statements including the title.

Comment 1.2*: The resolution of most Figures in the reviewer PDF is poor. In Figure 4B, for example, it isn't possible to assess co-labeling by EdU/BrdU. Would it be possible to provide in*

the supplement some high-res, single confocal slices? If I got it right, 12.6% of the blastema cells were double positive. How many cells were double positive in the tentacle bud? Was it a similar percentage?

Response 1.2

The poor resolution of figures is probably due to the size limitation of the uploaded files, and these issues will be solved when submitting the final version that allows high resolution.

As requested, for Figure 4B, we have added a magnified image of single confocal slices to allow the evaluation of EdU and BrdU co-staining (Revised Fig. 4C). We have also quantified the number of EdU^+ & $BrdU^+$ (double positive) cells in the bulb region to clearly convey the results of the image analysis. In blastema, $BrdU^+$ repair-specific proliferative cells did not often merge with EdU^+ cells, which were labeled as resident stem cells (average 13.6%), whereas in the bulb, many $BrdU^+$ cells merged with EdU (average 68.7%) (Revised Fig. 4C-4D). These results suggest that resident stem cells in the bulb do not actively migrate to the injury site during the 24 hours after amputation. This also supports our main conclusion that resident stem cells and repair-specific proliferating cells in blastema are of different cellular origin with different characteristics.

Comment 1.3*: Usage of 'ectoderm' and 'endoderm' to denote adult cnidarian external and internal epithelial tissues, respectively, is misleading. Ectoderm and endoderm are embryonic lineages that give rise to specific tissues in the adult. There is no evidence in the literature from any cnidarian showing that their external tissue derives exclusively from their embryonic ectoderm and/or that their gastric tissue derives exclusively from endoderm. Use 'epidermis' and 'gastrodermis', or some other term that does not refer to embryonic origin, instead.*

Response 1.3

We appreciate the reviewer for this constructive comment. We have changed the terms ‘ectoderm’ and ‘endoderm’ to ‘epidermis’ and ‘gastrodermis,’ respectively, in the revised manuscript.

Comment 1.4*: Line 338. Nematocyte clusters are not visible on Figure 1D III.*

Response 1.4

As we had not clearly defined nematocyte clusters, we have defined nematocyte clusters in

tentacles with different resolutions in the revised Figure S1A. Both stereo and confocal microscope images show that the spherical structures are repeatedly formed on the tentacles (Fig S1Aii, S1Aiii), and they are stained with poly-γ-glutamate, which is a mature nematocyte marker, indicating that these spherical structures correspond to nematocyte clusters (Fig S1Aiii'-S1Aiii"). Based on this criteria, Figure 1D (iii) shows a spherical bulge formed at the tip of the regenerating tentacle, indicating the newly formed nematocyte cluster. In order to clearly convey our intention, we have revised the manuscript as follows:

> (Revised Manuscript; Page 13, Line 431-434)
> "Subsequently, tentacles began to elongate distally, and nematocyte clusters, the spherical structures stained with the mature nematocyte marker poly-γ-glutamate, formed at the tip of the regenerating tentacle at 48 hpa (Figs 1Diii, 1Fiii, and S1A)."

Comment 1.5*: Line 412-413. "24 hpa after amputation": "after amputation" is redundant.*

Response 1.5

As suggested, we removed "after amputation" from the manuscript as follows:

> (Revised Manuscript; Page 15, Line 515-516)
> "Together, these observations suggest that cell proliferation is accelerated near the injury site at 24 hpa."

Comment 1.6*: Studied cnidarians have two Piwi-like genes. Which one has been studied here? Regarding Nanos genes, some are Nematoblast rather than i-cell markers. Is Cladonema Nanos1 a likely i-cell marker?*

Response 1.6

In contrast to some studied cnidarians [11,12], there is only one *Piwi* gene in *Cladonema pacificum*. The previous report by Hou et al. performed phylogenetic analysis to show the *Piwi* gene in *Cladonema pacificum* is homologous to the *Piwi1* gene in *Hydra vulgaris* (Hydra vulgaris Piwi AFQ20832.1) and *Podocoryna carnea* (Podocoryna carnea Piwi AAS01181.1) [13]. We thus used the *Cladonema Piwi* as a *Piwi1* homologue in this study.

Regarding *Nanos*, there are two *Nanos* genes in *Cladonema*, *Nanos1* and *Nanos2*, and we have utilized *Nanos1* as a stem cell marker and *Nanos2* as a nematoblast marker for the

following reasons.

While *Nanos1* has been regarded as a stem cell, or i-cell, marker in hydrozoans [14–16], *Nanos2* has been reported to be expressed in nematoblasts in *Hydractinia* [17]. We have examined *Nanos2* expression pattern in *Cladonema* medusa by FISH (Revised Fig. S3B, S3E, and S3F) and found that *Nanos2* is co-expressed with *Mcol1* (*Minicollagen 1*), which is a well-known nematoblast marker (Revised Fig. S3F) [18–20]. These results suggest that *Nanos2* in *Cladonema* is also expected to be a nematoblast marker.

There are four lines of evidence supporting that *Cladonema Nanos1* is a stem cell, or i-cell marker. First, *Nanos1* has been employed as a marker for stem cells in various hydrozoan models including *Hydra vulgaris* and *Clytia hemisphaerica* [14,21]. Second, *Nanos1* is also predicted to be a stem cell marker in *Cladonema* medusa because *Nanos1* is expressed at the branching site of the tentacles, in which more than 80% of the cells exhibit a stem cell morphology as shown in the previous work by Hou et al. [13]. Third, we have compared the expression pattern of *Nanos1* with other stem cell markers, *Piwi* and *Vasa1,* by FISH and found that *Nanos1* was localized in the bulb and branching sites of tentacles along with *Piwi* and *Vasa1* (Revised Fig. 3C, S3A-S3D, and S4A, S4B), suggesting that *Nanos1* is one of the markers for stem cells. Fourth, double FISH showed that *Nanos1* did not merge with *Mcol1* or *Nanos2*, both of which are nematoblast markers (Revised Fig. S3E, see also the following paragraph). These findings strongly suggest that *Nanos1* is an i-cell marker that can label part of stem cell populations. We have added the following sentence to the text in the revised manuscript.

> (Revised Manuscript; Page 16, Line 560-563)
> "Note that, while *Cladonema* possess two *Nanos* genes, *Nanos1* and *Nanos2, Nanos2*$^+$ cells co-express a nematoblast marker *Mcol1*, suggesting that *Nanos2* is a nematoblast marker rather than a stem cell marker in *Cladonema* medusa (S3E and S3F Fig), similar to *Hydractinia* [47]."

Comment 1.*7: Line 421. No stem-like cells are shown in Figure 2. It only shows S-phase cells; some may be committed progeny rather than stem cells (see also comment 1C). If the authors wish to identify the nature of S-phase cells, they should show EdU incorporation in conjunction with an i-cell marker like Piwi1/2; they do show it in Figure 3E (with Nanon1) where many if not most Nanos1+ cells are EdU negative.*

Response 1.7

In Figure 2, we intended to show the distribution pattern of proliferative cells, not stem-like cells, and that is why we showed only S-phase cells by EdU pulse labeling. Our results indicate that highly proliferative cells accumulated at the injury site (Revised Fig. 2Aii) and that cell proliferation is indeed more active at the amputation site than the bulb where resident stem cells are present (Revised Fig. 2D-2F), which together formed the foundation for further addressing cellular types and the role of blastema during tentacle regeneration. The cell types in blastema and their stem-like characteristics are described in detail in the revised Figure 3 and Figure S4.

Comment 1.8*: Line 628. Not all hydrozoan i-cells are pluripotent. Hydra i-cells are multipotent (the authors mention this in line 634).*

Response 1.8

Indeed, not all hydrozoan i-cells exhibit pluripotency. Consequently, in the revised manuscript, we have modified the term "pluripotent" to "pluripotent or multipotent" (please see page 22, line 781).

Comment 1.9*: Line 635. Watanabe et al. is not in the ref list.*

Response 1.9

Thank you for noticing the reference omission. We have added the 'Watanabe et al.' to the reference list.

Comment 1.10*: Line 723. Replace "homologous" by "similar".*

Response 1.10

We have replaced the term "homologous" with "similar" in line 723 of the original manuscript, which corresponds to line 892 in the revised manuscript.

<u>Response to Reviewer #2</u>

Comment 2.1*:*

Fujita et al investigated the cellular mechanisms of tentacle regeneration in the hydrozoan medusa Cladonema pacificum. They demonstrate that after tentacle ablation, highly

proliferative cells quickly accumulate at the site of injury forming a blastema. Through a combination of approaches, they further demonstrate that those blastema cells are (i) not derived from resident stem cells (i-cells) bur appear locally after damage; and (ii) are the principal cellular source of the epithelium in the newly formed tentacle, while resident i-cells contribute to nematogenesis and tissue elongation during both homeostasis and regeneration. Overall, I find the experiments well designed and executed, the manuscript well elaborated and the conclusions solid - actually I don't have request for additional experiments that would be needed to strengthen the conclusions. However, I am less convinced about the complete novelty of the findings, in particular of the central claim, that this "work is the first to demonstrate that repair-specific proliferative cells appear upon injury in cnidarians". As waves of proliferation of epithelial cells at the site of injury were shown to happen in several cnidarians (notably Hydra, Hydractinia, Clytia and Nematostella), it is not so surprising that those proliferating cells are not only migrating cells but also local epithelial cells re-entering the cell cycle. It remains an interesting paper, providing further information on an additional cnidarian species.

Response 2.1

We thank the reviewer #2 for acknowledging the impact and quality of our work.

Regarding the novelty of our findings, we have shown that most repair-specific proliferative cells (RSPCs) in blastema during the *Cladonema* tentacle regeneration are not derived from resident stem cells while exhibiting i-cell morphological characteristics (Revised Fig. 3, 4, and S5-S7). We have further found that RSPCs express the stem cell markers *Nanos1, Piwi,* and *Vasa1* with heterogeneous expression patterns among these cells, suggesting that RSPCs are heterogeneous stem-like cell population in terms of gene expression and likely their cellular characteristics (Revised Fig. 3Eiii, 3Eiv, 3F and S4C-S4E). Most importantly, we have demonstrated that the majority of RSPCs differentiate into epithelial cells in the newly regenerated tentacle whereas some RSPCs can still give rise to neurons (Revised Fig. S10), suggesting that RSPCs are multipotent, or at least bipotent, stem cells that preferentially generate epithelial cells. These results stand in stark contrast to the previous reports that describe pluripotent i-cells migrating to the injury site [9] or stem-like epithelial cells directly contributing to whole-body regeneration [22,23].

As suggested by the reviewers, studies using polyps like *Hydractinia* and *Hydra* have shown the existence of proliferating epithelial cells. During head regeneration of *Hydractinia*, cell proliferation occurs at the injury site. Although small numbers of epithelial cells can proliferate, more than 90% of proliferative cells are i-cells that migrate from the body column [9], which is totally different from *Cladonema* tentacle regeneration where RSPCs contribute to blastema. During head regeneration of *Hydra* after mid-gastric amputation, apoptosis occurs at

the injury site, and the following cell proliferation is observed in the area immediately below the amputation site [24]. In this case, most of these proliferating cells are indeed i-cells, and epithelial cells are rare. Intriguingly, when i-cell lineages (i-cells, neurons, nematocytes) are removed from *Hydra*, epithelial cells transiently proliferate at 18-30 hpa, and head regeneration seems to occur normally [22,23]. It is noteworthy that the *Hydra* epithelial lineages are self-renewing proliferative cells while their morphology and characteristics are distinct from i-cells [25], suggesting that epithelial cells in *Hydra* are not terminally differentiated cells, but rather stem cells, which may be a special feature of *Hydra* that is not conserved in other cnidarians. In contrast to the reviewer's understanding, regarding the sea anemone *Nematostella* and the jellyfish *Clytia*, existing literature lacks a definitive characterization of epithelial cell division after injury [16,26–29], although these reports showed the requirement for cell proliferation and the involvement of stem cells during regeneration.

Taken together, our study is pioneering as it represents the first clear demonstration that RSPCs appear as a different population from resident stem cells and preferentially differentiate into epithelial cells during appendage regeneration. We have added the following sentence in the discussion:

> (Revised Manuscript; Page 23, Line 810-813)
> "While previous studies using other cnidarians have described active cell proliferation upon injuries, these proliferating cells are pluri/multipotent stem cells migrating to the injury [20,21,27,62], not lineage-restricted stem/progenitor cells like RSPCs."

<u>Response to Reviewer 3</u>

In this manuscript, Fujita et al. describe the process of blastema formation in the medusa jellyfish Cladonema pacificum. Using nucleoside-based lineage tracing techniques, the authors show the existence of two distinct subpopulations of proliferative cells that differentially contribute to tentacle regeneration - "homeostatic proliferative cells", which contribute to general tissue turnover as well as regeneration, and "repair-specific proliferative cells", which emerge only upon amputation. Each population contributes to the differentiation of new cell types during regeneration; repair-specific cells yield only epithelial cells, whereas homeostatic cells generating nematocytes, epithelial cells, and new stem cells. Based on irradiation experiments, the authors propose that repair-specific proliferative cells do not descend from i-cells (Cnidarian stem cells), but rather emerge de novo upon injury.

While this paper certainly demonstrates the presence of multiple proliferative cell populations, the data supporting the major claims are sometimes inconclusive and over-

interpreted. There are a number of major comments that need to be addressed and include inconsistencies throughout the paper, experimental problems involving irradiation, and interpretations of data. The findings presented here contribute to the growing world of invertebrate stem cell biology, but to convincingly support their claims the authors must resolve these concerns.

Major comments:

Comment 3.1*: Inconsistency of data throughout the paper*

Throughout the paper, the authors use thymidine analogs (BrdU and EdU) as lineage tracing tools. Consistent with their role as homeostatic stem cells, a short pulse with EdU labels i-cells in intact tentacles. Why does EdU incorporation look dramatically different in Figure 2A compared to Figure S4A? Both figure legends indicate a 1h pulse labeling, and yet the data show opposite results. This is an essential experiment because it provides one of the biggest pieces of evidence for the authors' claim of distinct cell populations; clarity is therefore necessary. Adding schematics for each pulse chase experiment will help this effort.

Response 3.1

In the original manuscript, the EdU images of 24-72 hpa were shown after 1 h incubation with EdU 20 μM, which was a different condition from the other experiments (e.g. revised Figure S5A). We thus replaced these images with the results of 1 h incubation with EdU 150μM (Revised Fig. 2A). In this condition, strong EdU signals were observed near the injury site at 24 hpa while relatively weak but recognizable EdU signals were detected in the bulb region of both intact and 24 hpa samples (please see Fig. 2A(i) and (ii)).

To clarify each pulse chase experiment using EdU and/or BrdU, we have added experimental schematics to the figures in the revised manuscript (Revised Fig. 4-6, S2, S4-S8, S10-S11).

Comment 3.2*: To demonstrate the contribution of these proliferative subpopulations to different lineages, the authors perform EdU labeling in combination with FISH for the cell-type specific markers Nowa and Mcol1 (Figure S6). Despite showing the same time points for both markers, however, the EdU incorporation looks vastly different. Is such variation normal across experiments? Why is this not addressed?*

Response 3.2

The expression level of *Mcol1* was extremely high, and indeed the FISH *Mcol1* signal (Cy5)

invaded the channel for EdU signal (Alexa Fluor 488). We thus adjusted the intensity of EdU to become darker, resulting in a different view than appears in other figures. In order to clarify the EdU signals, we have added the magnified figures and single slice images (Revised Fig. S8A). Notably, from *Mcol1* FISH experiment with EdU labeling, we confirmed that EdU 24 h incubation labeled more *Mcol1* cells than EdU 1h incubation (Revised Fig. S8A) although the ratio of EdU positive cells was similar to Revised Figure 5i wherein EdU 24 h incorporation was performed together with BrdU (Fig. R2). These results suggest that there is no significant difference of EdU incorporation across experiments.

Regarding *Nowa* FISH experiments, there was an error in the sequence that was used to design the FISH probe, so we have removed the corresponding data from the revised manuscript.

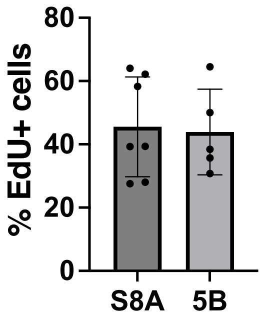

Fig. R2: Rate of EdU$^+$ cells in the bulb. EdU (150 μM/24 h) labeling from Figure 5B and Figure S8A. Quantification was performed on a single slice of each confocal image. The quantification area is 35 μm^2.

Comment 3.3.1*: Except for the qPCR in FigureS5A, all the quantifications in this paper are derived from microscopy images. While this is not a problem in and of itself, it does mean that the representative images chosen to accompany graphs need to be convincing. Unfortunately, there is repeated inconsistency throughout the paper between quantifications and the representative images.*

Response 3.3.1

Due to the size limitation of the uploaded files, the quality of the figures might not provide sufficient quality, and some figures did not clearly show whether the signals were merged or not. To address the image issue, we have added enlarged images particularly for the quantified area as well as the size of the quantified area to figure legends (e.g. Revised Fig. 3E and 4C). In addition, to avoid inconsistency between quantifications and representative images, we have replaced some images that were less convincing (e.g. Revised Fig. 3E, 4C, 5B and 6B).

Comment 3.3.2*: The authors state that, at 24hpa, only 12.6% of EdU+ cells were also expressing BrdU. The images shown (Figure 4Bi, i', and i'') do not support this claim. Better representative images, and perhaps an overlay of the BrdU/EdU channels at this time point are needed.*

Response 3.3.2

For clarity, we have added EdU/BrdU magnified images of single confocal slices (Revised Fig. 4Ci, 4Cii) and further quantified the ratio of EdU^+ to $BrdU^+$ cells in both bulb and blastema (Revised Fig. 4D). The quantification showed that the $EdU^+/BrdU^+$ cell ratio is 13.60±2.18% in blastema, although it is 68.66 ± 4.82% in the bulb (Revised Fig. 4D). This result suggests that the proliferating cells in the bulb region at 24 hpa are still resident stem cells (resident homeostatic stem cells, RHSCs), but most blastema cells (repair-specific proliferative cells, RSPCs) are derived from a different lineage. We have changed the following sentence in the text:

> (Revised Manuscript; Page 18, Line 622-624)
> "Importantly, only 13.6% of $BrdU^+$ RSPCs were EdU^+ in blastema, supporting the notion that the migration of RHSCs may not be sufficient to form blastema (Fig 4Bi, 4Cii, and 4D)."

Comment 3.3.3*: In Figure 3, the Nanos1+/EdU+ cell images in E do not corroborate the quantifiations in F. Including a zoomed in image of the double positive cells would better support the claim, as the current images are impossible to interpret.*

Response 3.3.3

To address the reviewer's concern, we have added enlarged images (Revised Fig. 3Eii and 3Eiv) that corroborate the quantification of *Nanos1*$^+$/EdU^+ cells (please see the revised Fig. 3E, 3F). This result shows that the majority of EdU^+ cells (69.7%) co-express *Nanos1* in the bulb whereas fewer EdU^+ cells (30.3%) co-express *Nanos1* in blastema, supporting our proposal that resident homeostatic stem cells and repair-specific proliferative cells are of different lineages.

Comment 3.3.4*: Figure S4E shows a 2-fold increase in the number of EdU+ cells, but the*

images in panel D do not support these numbers.

Response 3.3.4

To clarify the image issue, we prepared single channel images of EdU for Figure S4E in the original manuscript (Revised Fig. S5C). Based on these images, we counted the number of EdU^+ cells in the whole tentacle at 24 hpa (Revised Fig. S5Ciii, S5D) as well as EdU^+ cells in intact tentacle (0h and 24 h no-amputation; Revised Fig. S5Ci, S5Cii, S5D). The size of the area where we counted EdU^+ cells in intact tentacle was similar to that of the regenerating tentacles at 24 hpa (Revised Fig. S5E), suggesting that following EdU incorporation, the number of EdU^+ cells increased similarly over 24 h in both no-amputation and regenerating tentacles. The quantification method and the average area of the quantified area were added to the figure legend as follows:

> (Revised Manuscript; Page 40, Line 1392-1396)
> "(D) The number of EdU^+ cells labeled before amputation. Counted area is the whole regenerating tentacle at 24 hpa. Detailed information is in (E). Intact: n = 6 (tentacles), 24 h (no amp): n = 6, 24 hpa: n = 6. (E) Area size used for quantification. Note that the area of quantification at 24 hpa is similar."

Comment 3.3.5*: Figure S5C claims that the % Nanos1+ cells decrease upon irradiation, but the image in panel B instead seems to show a redistribution towards the middle of the image. Were these cells not counted? How were the % of Nanos1+ cells quantified?*

Response 3.3.5

In the original manuscript, we did not count $Nanos1^+$ cells in the middle area, and instead we counted $Nanos1^+$ cells only in the tentacle bulb. To address the reviewer's concern, we have counted the number of $Nanos1^+$ cells in the whole tentacle including the middle area (Revised Fig. S7A-S7C) and confirmed that after X-ray irradiation, $Nanos1^+$ cells decreased in a dose-dependent manner (Revised Fig. S7B-S7C).

Regarding the redistribution phenotype of $Nanos1^+$ cells, indeed some $Nanos1^+$ cells seemed to show redistribution to the middle area (Revised Fig. S7Bii: 30 Gy and iii: 50 Gy). These surviving $Nanos1^+$ cells may change localization as a response to irradiation, but at the current stage we have not pursued the cause as this topic is beyond this work's scope. More importantly, we have focused on the condition of 75 Gy X-ray irradiation for the subsequent experiments that address blastema formation after elimination of $Nanos1^+$ cells (Revised Figure

4H-4J).

Comment 3.3.6: *In lines 416-418, the authors state that "In the regenerating tip, the number of PH3+ an EdU+ cells was about 4 and 20 times greater, respectively, than the corresponding area in the intact tentacle" and refer to Figures 2D-2F. Are the authors comparing Bulb (a) to Bulb (b)? If so, these numbers would be 0 and ~1.5. Are they comparing Bulb (a) to Blastema? If so, the numbers would be ~3.3 and 3. Where are these numbers in the text coming from?*

Response 3.3.6

In lines 416-418 in the original manuscript, we intended to state that the rate of EdU^+ cells and $PH3^+$ cells in blastema increased 20- and 4-fold compared to the amputation site. This difference is due to the quantification that the rate of EdU^+ cells is 2.13% at the amputation site and 46.5% in blastema (~20 fold), while the rate of $PH3^+$ cells is 0.70% at the amputation site and 2.90% in blastema (~4 fold). We have changed the description as follows:

> (Revised Manuscript; Page 15, Line 519-522)
> "In the regenerating tip, the number of $PH3^+$ and EdU^+ cells was about 4 and 20 times greater, respectively, than the corresponding area (amputation site) in the intact tentacle (Fig 2D-2F)."

Comment 3.4: *Irradiation experiments*

The authors rely on X-ray irradiation for multiple experiments, including to demonstrate that the repair-specific proliferative cells do not derive from i-cells. However, the level of irradiation seems insufficient to support their claim. The elimination of i-cells after irradiation was supposedly confirmed by assessing levels of Nanos1 mRNA (Figure S5A), but Nanos1+ cells are still markedly present at 3dpi (Figure S5B). This may be another instance of non-representative images (see above), but regardless, the data are not convincing. If the irradiation is not sufficient to remove all of the stem cells prior to amputation, the experiments in Figure 4F-H are meaningless. The authors therefore need to increase the amount of irradiation and fully eliminate the homeostatic proliferating cells in order to support their claim that the "repair-specific proliferative cells" do not derive from i-cells. The experiments in Figure 5 that rely on irradiation will need to be repeated as well with a higher dose. This is an essential component of the paper, and must be addressed to support the claim of two separate proliferative populations.

Response 3.4

We thank the reviewer for this critical comment. To address the concern, we identified a condition that can remove almost all *Nanos1*$^+$ and found that 75 Gy X-ray irradiation caused loss of *Nanos1*$^+$ cells in the tentacle (Revised Figure S7A-S7C, S7F, and S7G). In order to eliminate these *Nanos1*$^+$cells by other methods without X-ray irradiation, we treated *Cladonema* medusae with hydroxyurea (HU) or Mitomycin C and confirmed that *Nanos1*$^+$ cells were successfully removed (Revised Figure S7D-S7G). Notably, even in these conditions where most *Nanos1*$^+$ cells were eliminated, we detected repair-specific proliferating cells (Revised Fig. 4H-4J), further supporting our claim that repair-specific proliferative cells do not derive from resident i-cells in the bulb. Please also refer to **Response 1.1.7** regarding the details and rationale of the experiments.

Comment 3.5*: To target the "repair-specific proliferative cells" during regeneration, the authors used UVA irradiation to spatially target the blastema. This radiation yielded a slight decrease in the number of EdU+ cells and showed a significant inhibition of regeneration, but these data do not indicate a population-specific effect. To fully support a claim for functional specificity of these populations, further validation needs to be shown. For example, even if tentacles do not form after irradiation (Figure S8C), are there still new epithelial cells coming from the homeostatic proliferative cells (as shown in Figure 5)?*

Response 3.5

To clarify the effect of UVA exposure on repair-specific proliferative cells, we have performed additional experiments (Revised Fig. 6D-6F). We first confirmed that UVA radiation significantly suppressed the proliferation of repair-specific proliferative cells in blastema at 48-72 hpa, in addition to comparisons between non-UV exposure controls and irradiated samples (Revised Fig. 6D-6F).

In order to determine any effects of UV exposure on resident homeostatic stem cells (RHSCs) in the bulb, we have quantified the proliferation of RHSCs after EdU pulse labeling and found that the increase of RHSCs was similar to the non-UV exposure controls (Revised Fig. S11C-S11D). To further bolster this line of evidence, we have investigated the timing of nematocyte cluster formation, which is the descendent of RHSCs (Revised Fig. S11E-S11G). Consistent with the observation that RHSCs' proliferative capacity is not impaired (Revised Fig. S11C-S11D), nematocyte clusters formed mainly at 48-72 hpa in the UVA-irradiated tentacles, which was similar to controls (Revised Fig. S11E-S11G). These results suggest that blastema

exposure to UVA has no significant impacts on RHSCs. We have added the following sentence to the text in the revised manuscript.

> (Revised Manuscript; Page 22, Line 756-759)
> "In contrast, we confirmed that cell proliferation and the timing of nematocyte cluster formation by RHSCs were similar to the non-UV exposure controls, suggesting a minimal impact of UV laser exposure on RHSCs (S11 Fig)."

Comment 3.6*: Overinterpretation of lineage tracing results*
The major claim of this paper is the description of two functionally distinct proliferative cell populations, and this conclusion is primarily drawn from the dual BrdU/EdU labeling experiments. The authors labeled the "homeostatic proliferative cells", aka i-cells, with a short pulse of EdU prior to amputation. The "repair-specific proliferative cells", of which the authors do not know the origin, were then labeled with BrdU at 24hpa. While there is indeed a difference in BrdU and EdU incorporation in the regenerating tentacle, I find the claim that the "repair-specific proliferative cells" emerge de novo premature. Instead, a more likely hypothesis is that an injury-responsive reserve population of i-cells exists. Interestingly, the authors mention this phenomenon in the Discussion (lines 667-678) and include a mention in Figure 6B, yet seem to repeatedly dismiss the possibility in the text in favor of a de novo hypothesis (lines 162-164, 485-488, 729-733). Unless the authors can rule out that the "repair-specific proliferative cells" are not simply a slow-cycling subpopulation of i-cells, the conclusions need to be significantly reworded.

Response 3.6
We agree with the reviewer that our claim that the "repair-specific proliferative cells (RSPCs)" emerge *de novo* is premature because so far there is no clear evidence to support where exactly RSPCs come from. We thus have avoided the term "appear *de novo*" and instead focused on the discovery of two functionally distinct stem/proliferative cell populations 'resident homeostatic stem cells (RHSCs)' and 'RSPCs'. We have revised the following sentence in the text:

> (Revised Manuscript; Page 18, Line 611-614)
> "…one population are the "resident homeostatic stem cells (RHSCs)," composed of i-cells localized at the bulb, and the other are "repair-specific proliferative cells (RSPCs)" that appear as blastema upon amputation."

Comment 3.7*: The DiI injection experiments (Figure S4B-C) are inconclusive. There is no evidence that the injected dye is labeling stem cells, so the fact that the labeled cells are not moving during regeneration is irrelevant. The authors need to either show that the DiI injection is specifically marking proliferative cells (for example, include FISH for a stem cell marker) or remove the experiment from the paper.*

Response 3.7

To address the concern associated with DiI injection experiments, we have examined the cell types of DiI labeled cells by incorporating EdU staining or β-catenin antibody staining. We confirmed that more than 75% of proliferating/stem cells are indeed labeled by DiI injection (Revised Fig. S6A-S6C). Please also refer to **Response 1.1.8** for details.

Comment 3.8*: Characterization of i-cells and blastema*

Throughout the text, the authors refer to the blastema as "undifferentiated cells with mitotic capacity that appear after damage such as amputation or irradiation" (lines 72-72). However, this description is not consistent with the standard definition in the field, which identifies a blastema simply as an unpigmented bud of cells near the site of an injury (Reddien and Sanchez-Alvarado, 2004). Mitotic cells are necessary for the maintenance and growth of a blastema, but not all cells within a blastema must be mitotic.

Response 3.8

There are several different definitions of blastema, depending on the model system and its regenerative strategy. For example, in amphibians, blastema is described as an unpigmented outgrowth consisting of a mass of undifferentiated progenitor cells near injury site [30,31]. In contrast, in planarians, blastema is defined as post-mitotic progeny of stem cells that differentiate to re-form the lost tissue [32]. Due to the lack of consensus about the term 'blastema' in the regeneration field, it is reasonable to refer to specific cell populations 'blastema' as long as they constitute the cellular source of newly building bodies and organs. It is true that not all cells in blastema are mitotic. In this study, we have thus defined blastema as follows:

> (Revised Manuscript; Page 3, Line 82-84).
> "Blastema can be defined as an undifferentiated cellular mass that contains cells with mitotic capacity and appears after damage such as amputation [33,34].."

Comment 3.9: *Cell types within the blastema were characterized using a antibody against B-catenin (Figure 3, Figure S7) or aPKC (Figure S7), which the authors state can distinguish between stem cells, nematocytes, and epithelial cells. Are these the only three potential cell types? What about muscle and neurons?*

Response 3.9

In cnidarians, muscle cells are considered to be 'epitheliomuscular cells' (see the schematic from [35] below); as such, cell types identified by β-catenin or aPKC antibody must include muscle cells.

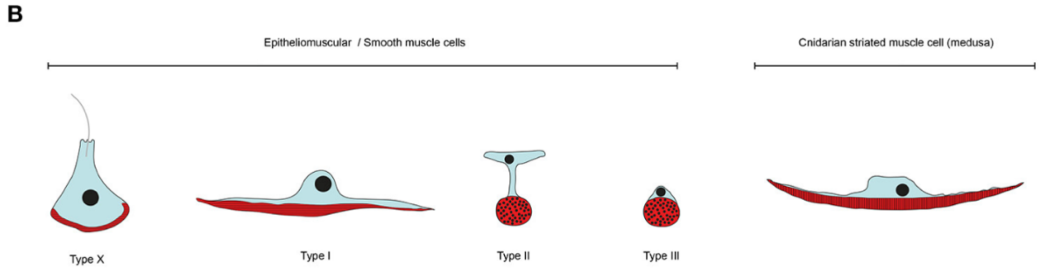

We have added the description that epithelial cells are epitheliomuscular cells in the revised manuscript as follows:

> (Revised Manuscript; Page 5, Line 159-161)
> "The *Cladonema* medusa tentacle is primarily composed of bi-layered epithelial tissues (epidermis and gastrodermis) that include muscle fiber, also called epitheliomuscular cells [23]"

Regarding neurons, it is difficult to distinguish their origin by immunostaining with β-catenin or aPKC antibodies; this is due to the fact that the cell bodies of neurons are mainly distributed in the bulb around the eye spot, and are less frequently localized in the apical side of the tentacle where these antibody signals are available. Instead of using β-catenin or aPKC antibodies, we have combined EdU-labeled tracking with antibody staining of a neuronal marker, FMRFamide (Revised Fig. S10). With this method, we have successfully confirmed that neurons in the regenerating tentacle are derived from both repair-specific proliferative cells (RSPCs) and resident homeostatic stem cells (RHSCs) (Revised Fig. S10). While both RSPCs and RHSCs can produces neurons, RHSCs give rise to neurons more frequently than RSPCs (Revised Fig.

S10E), likely reflecting the different properties of the two cell populations. We've included these allusions to these results in the revised text as follows:

> (Revised Manuscript; Page 20, Line 702-704)
> "Intriguingly, we also noticed that EdU-labeled RHSCs merged with FMRFamide$^+$ neurons (S10A and S10B Fig), suggesting that RHSCs can differentiate into neurons during tentacle regeneration."
>
> (Revised Manuscript; Page 21, Line 739-743)
> "Of note, we found that a small portion of EdU-labeled RSPCs merged with FMRFamide$^+$ neurons, although these populations are significantly smaller than the RHSCs (S10C-S10E Fig). Combined, these findings indicate that RSPCs are multipotent, or at least bipotent, stem cells that preferentially generate epithelial cells during regeneration."

Comment 3.10*: If Nanos and Piwi1 are truly markers of i-cells, the data shown in Figure 3C and S3C are inconsistent with the claim that 80% of the cells in the blastema are i-cells. Further validation of these cell lineages using fluorescent in situ hybridization is necessary to convincingly characterize the composition of the blastema. Furthermore, why aren't all EdU+ cells Nanos+ positive (Figure 3E-F)?*

Response 3.10

Considering the fact that most of the blastema cells exhibit i-cell morphology (Revised Fig. 3A-3B) while only some express markers for i-cells such as *Nanos 1* and *Piwi*, it's possible that these stem cell populations are heterogenous, and there is no single maker for stem cells. To test the possibility, we have investigated the expression pattern of stem cell markers in repair-specific proliferative cells (RSPCs) in detail. In addition to *Nanos1* and *Piwi* [13], we have also cloned another stem cell marker *Cladonema Vasa1* and examined its expression by FISH. While these stem cell marker positive cells were accumulated in blastema in the regenerating tentacle, none of the markers were able to stain the majority of RSPCs in blastema (Revised Fig. 3Eii, 3F and S4C-S4E; *Nanos1: 30.3 %, Piwi: 36.8%, Vasa1: 17.2%*). Although we have also tried to examine co-localization of these stem cell marker genes (e.g. *Nanos1* and *Piwi*) with double FISH, we were not able to clearly detect these genes' expression. This is most likely due to the fact that gene expression of stem cell marker genes is not very high, as we successfully detected expression of two genes with double FISH when we examined nematocyte marker genes

(*Mcol1* or *Nanos2*) together with *Nanos1* (Fig. S3E, S3F).

In addition to heterogenous expression of marker genes, the blastema cells more preferentially differentiate into epithelial cells relative to resident stem cells (Revised Fig. 5E-5G and 6A-6C), suggesting that the molecular and cellular characteristics of RSPCs as stem/progenitor cells are different between the two stem-like cell populations. These observations strongly support the possibility that RSPCs constitute a distinct cellular population from resident stem cells, and this is probably one of the reasons why all RSPCs or EdU$^+$ cells in the bulb do not show the single i-cell marker genes like *Nanos1*.

Minor comments:

Comment 3.11*: Inconsistent terminology*

The authors flip-flop between using "i-cell" vs. "resident stem cell" vs "stem-like cell" throughout the text. Is this because there is no way to determine which cells are truly stem cells? Continuing this trend of ambiguity, the authors dance around the question of cell origin by labeling subpopulations of cells as "homeostatic" vs "repair-specific" vs "injury-responsive". These phrases should be more consistent to aid in clarity while reading the manuscript.

Response 3.11

In hydrozoan species including *Hydra*, *Hydractinian*, and *Clytia,* i-cells have been recognized as resident stem cells, and *Nanos1*, *Piwi1*, and *Vasa1*, which are also known as germline genes, have been utilized as i-cell markers [14]. In the *Cladonema* medusa tentacle, for example, these i-cell marker genes are expressed in the bulb, and i-cells in the bulb behave as resident stem cells (Revised Fig. 3, S3 and S4) [13]. In this work, we also initially referred to i-cells in the bulb as resident stem cells, but we discovered that i-cell marker genes as well as i-cell morphology are common features for proliferating cells in blastema after amputation of the tentacle (Revised Fig. 3, S3, and S4).

To distinguish two cell populations with i-cell characteristics, based on the result of lineage tracing, we have defined i-cells in the bulbs as ‘resident homeostatic stem cells (RHSCs)’ since they differentiate into most cell lineages including epithelial cells, nematocytes, and neurons (Revised Fig. 5A-5G and S10A-10B) both in homeostasis and regeneration. By contrast, we have defined proliferative cells in blastema as those that preferentially differentiate into epithelial cells but not nematocytes, while some can become neurons in the regenerating tentacle as ‘repair-specific proliferative cells (RSPCs)’. In order to make the terms consistent, we refer to them uniformly as such after establishing that RHSCs and RSPCs are derived from different cellular sources. We have introduced these two cell populations as follows:

(Revised Manuscript; Page 18, Line 611-614)
"…one population are the "resident homeostatic stem cells (RHSCs)," composed of i-cells localized at the bulb, and the other are "repair-specific proliferative cells (RSPCs)" that appear as blastema upon amputation."

Comment 3.12*: Figure organization and labeling*
Many of the figure images are difficult to interpret, and the authors would benefit from changing pseudocolors and/or providing single-channel images. The organization of figures is also often confusing, and panels are out of order.
Comment 3.12.1*: Figure 1Gii: Include FMRFamide only channel*

Response 3.12.1
We have added a FMRFamide only channel in Revised Fig. 1G.

Comment 3.12.2*: Figure 2A: Change pseudocolors, red is difficult to see on top of the green channel*

Response 3.12.2
In the original Figure 2A, we actually used magenta for EdU signal and green for DNA as pseudocolors, as we have used the same pseudocolor combination throughout the manuscript (see Revised Fig. 2G-2I and 4I, for example). We realized the issue in Figure 2A was more likely the intensity of the EdU signal due to the low concentration of EdU (EdU 20 μM, 1 hour), unlike other EdU incorporation experiments (EdU 150 μM, 1 hour). In the revised manuscript, we have performed the same experiments with 1 hour incubation of EdU 150 μM and replaced these images in Revised Fig. 2A. Importantly, EdU only channels were also shown below the pseudocolor images, which clearly indicates the distribution of EdU^{+} cells.

Comment 3.12.3*: Figure 2H-I: Include EdU only channel*

Response 3.12.3
We have added an EdU single channel in Revised Fig. 2G-2I.

Comment 3.12.4: *Figure 3E: Include separate channels for EdU and Nanos*

Response 3.12.4
We have added separate channels for EdU and *Nanos1* in Revised Fig. 3E.

Comment 3.12.5: *Figure 4G: Include EdU only channel*

Response 3.12.5
We have added an EdU only channel in Revised Fig. 4I.

Comment 3.12.6: *Figure 5: Lettering is confusing. Why is C in the top row above B, why are G and I above F, etc. Please re-order.*

Response 3.12.6
In principle, we arranged the panels in order from left to right, top to bottom, but we occasionally adjusted some of the panels to fit into the limited figure space. Regarding Figure 5, in the revised manuscript, we have arranged the panels such that the upper left panels are A-C, and the upper right panels are D-G. Indeed, we often see such arrangement of the panels in recent PLOS Biology papers. We also want to mention that the panel order issue is rather a production issue, not the content of the manuscript.

Comment 3.12.7: *Figure 5L: Please include zoomed-in photo of EdU+ cell types, current images are not sufficient.*

Response 3.12.7
We have added zoomed-in photos that can distinguish EdU+ cell types in Revised Fig. 6B.

Comment 3.12.8: *Figure S2B: The colors indicating basal/middle/distal are swapped*

Response 3.12.8
We have changed colors indicating basal/middle/distal in Revised Fig. S2A-S2B.

Comment 3.12.9*: Figure S3: Re-organize, C should not be below D/E.*

Response 3.12.9

We have re-organized the panels and solved this issue in the Revised Figure S3.

Comment 3.12.10*: Figure S3D: Include zoomed-in photo of double-positive cells for clarity.*

Response 3.12.10

We have obtained better resolution images for *Piwi*$^{+}$ and EdU^{+} double positive cells and added zoomed-in images in Revised Fig. S4C. In the revised manuscript, we have also characterized the localization of *Vasa1*$^{+}$ cells and similarly showed *Vasa1*$^{+}$ and EdU^{+} double positive cells, together with a zoomed-in image (Revised Fig. S4D). Quantification was further performed for these double positive cells (Revised Fig. S4E).

Comment 3.12.11*: Figure S4D: Include EdU only channel*

Response 3.12.11

We have added an EdU single channel in Revised Fig. S5C.

Comment 3.12.12*: Figure S5B: Include EdU only channel*

Response 3.12.12

The original Figure S5B showed DNA and *Nanos1* FISH staining after X-ray, and did not include an EdU channel. We have thus added the images of the *Nanos1* single channel in Revised Fig. S7B.

Comment 3.12.13*: Figure S6A/C: Include zoomed-in images of double-positive cells*

Response 3.12.13

As requested, we have added zoomed-in panels in Revised Fig. S8A.

Comment 3.12.14: *Figure S8C: Include EdU only channel*

Response 3.12.14

We have added an EdU single channel in Revised Fig. 6E.

Comment 3.12.15: *Quantifications*

Figure 2B/3B: Error bars should be added to demonstrate variability across samples

Response 3.12.15

We have added error bars in Revised Fig. 2B and 3B.

Comment 3.13: *Methods*

How were basal/middle/distal regions (Figures 2 and S2) determined? Was it by eye or did the authors quantify distances? This is not in the methods section but is repeatedly used for quantifications.

Response 3.13

We divided the tentacles into three equal parts by measuring the length and defined each region as basal, middle, and distal, from the basal side to the distal side. We have added descriptions to the methods section.

> (Revised Manuscript; Page 11, Line 374-378)
>
> "*Distribution of cells*
>
> Three areas were defined (the basal, middle, and distal regions) by dividing the tentacle into three equal lengths in confocal images (Figs 2A-2B, 3C-3D and S2A-S2B). The number of $PH3^+$, EdU^+, or $Nanos1^+$ cells were counted manually in each area using the multipoint tool of ImageJ/Fiji."

Comment 3.14: *Spelling/grammatical errors*

- Line 62: "Common regeneration processes" should be "common regenerative processes"

- Line 70: "may thus" should be "may therefore"

- Line 87: "intensively studied" should be "extensively studied"

- Line 123: "is prerequisite" should be "is a prerequisite"
- Figure 1G: "FMRFmide" should be "FMRFamide"
- Figure 3E-F: The images in E show "Intact" vs "24hpa", but the quantifications in F compare "Intact bulb" with "Blastema". Please keep the labels consistent.
- Figure 5C: "Epitelial" should be "Epithelial"

Response 3.14

We thank the reviewer for carefully reviewing in great detail and pointing out these issues. We have fixed the spelling and grammatical errors in the revised manuscript.

References

1. Daponte V, Tylzanowski P, Forlino A. Appendage Regeneration in Vertebrates: What Makes This Possible? Cells. 2021;10: 242. doi:10.3390/cells10020242
2. Pfefferli C, Jaźwińska A. The art of fin regeneration in zebrafish: The Art of Fin Regeneration. Regeneration. 2015;2: 72–83. doi:10.1002/reg2.33
3. Kikuchi K, Holdway JE, Werdich AA, Anderson RM, Fang Y, Egnaczyk GF, et al. Primary contribution to zebrafish heart regeneration by gata4+ cardiomyocytes. Nature. 2010;464: 601–605. doi:10.1038/nature08804
4. Bise T, Sallin P, Pfefferli C, Jaźwińska A. Multiple cryoinjuries modulate the efficiency of zebrafish heart regeneration. Sci Rep. 2020;10: 11551. doi:10.1038/s41598-020-68200-1
5. Stocum DL. Mechanisms of urodele limb regeneration. Regeneration. 2017;4: 159–200. doi:10.1002/reg2.92
6. McGann CJ, Odelberg SJ, Keating MT. Mammalian myotube dedifferentiation induced by newt regeneration extract. Proc Natl Acad Sci USA. 2001;98: 13699–13704. doi:10.1073/pnas.221297398
7. Salinas-Saavedra M, Febrimarsa, Krasovec G, Horkan HR, Baxevanis AD, Frank U. Senescence-induced cellular reprogramming drives cnidarian whole-body regeneration. Cell Reports. 2023;42: 112687. doi:10.1016/j.celrep.2023.112687
8. Bode HR, Scott Smith G. Regulation of interstitial cell differentiation inHydra attenuata: II. Correlation of the axial position of the interstitial cell with nematocyte differentiation. Wilhelm Roux' Archiv. 1977;181: 203–213. doi:10.1007/BF00848421
9. Bradshaw B, Thompson K, Frank U. Distinct mechanisms underlie oral vs aboral regeneration in the cnidarian Hydractinia echinata. eLife. 2015;4: e05506. doi:10.7554/eLife.05506
10. Govindasamy N, Murthy S, Ghanekar Y. Slow-cycling stem cells in hydra contribute to head regeneration. Biology Open. 2014;3: 1236–1244. doi:10.1242/bio.201410512

11. Juliano CE, Reich A, Liu N, Gotzfried J, Zhong M, Uman S, et al. PIWI proteins and PIWI-interacting RNAs function in Hydra somatic stem cells. Proceedings of the National Academy of Sciences. 2014;111: 337–342. doi:10.1073/pnas.1320965111

12. Lim RSM, Anand A, Nishimiya-Fujisawa C, Kobayashi S, Kai T. Analysis of Hydra PIWI proteins and piRNAs uncover early evolutionary origins of the piRNA pathway. Developmental Biology. 2014;386: 237–251. doi:10.1016/j.ydbio.2013.12.007

13. Hou S, Zhu J, Shibata S, Nakamoto A, Kumano G. Repetitive accumulation of interstitial cells generates the branched structure of *Cladonema* medusa tentacles. Development. 2021;148: dev199544. doi:10.1242/dev.199544

14. Leclère L, Jager M, Barreau C, Chang P, Le Guyader H, Manuel M, et al. Maternally localized germ plasm mRNAs and germ cell/stem cell formation in the cnidarian Clytia. Developmental Biology. 2012;364: 236–248. doi:10.1016/j.ydbio.2012.01.018

15. Klimovich A, Wittlieb J, Bosch TCG. Transgenesis in Hydra to characterize gene function and visualize cell behavior. Nat Protoc. 2019;14: 2069–2090. doi:10.1038/s41596-019-0173-3

16. Sinigaglia C, Peron S, Eichelbrenner J, Chevalier S, Steger J, Barreau C, et al. Pattern regulation in a regenerating jellyfish. eLife. 2020;9: e54868. doi:10.7554/eLife.54868

17. Kanska J, Frank U. New roles for Nanos in neural cell fate determination revealed by studies in a cnidarian. Development. 2013;140: e1608–e1608. doi:10.1242/dev.101246

18. Babonis LS, Martindale MQ. PaxA, but not PaxC, is required for cnidocyte development in the sea anemone Nematostella vectensis. EvoDevo. 2017;8: 14. doi:10.1186/s13227-017-0077-7

19. Zenkert C, Takahashi T, Diesner M-O, Özbek S. Morphological and Molecular Analysis of the Nematostella vectensis Cnidom. Dearden PK, editor. PLoS ONE. 2011;6: e22725. doi:10.1371/journal.pone.0022725

20. Adamczyk P, Zenkert C, Balasubramanian PG, Yamada S, Murakoshi S, Sugahara K, et al. A Non-sulfated Chondroitin Stabilizes Membrane Tubulation in Cnidarian Organelles. Journal of Biological Chemistry. 2010;285: 25613–25623. doi:10.1074/jbc.M110.107904

21. Mochizuki K, Sano H, Kobayashi S, Nishimiya-Fujisawa C, Fujisawa T. Expression and evolutionary conservation of nanos- related genes in Hydra. Development Genes and Evolution. 2000;210: 591–602. doi:10.1007/s004270000105

22. Sugiyama T, Fujisawa T. GENETIC ANALYSIS OF DEVELOPMENTAL MECHANISMS IN HYDRA. II. ISOLATION AND CHARACTERIZATION OF AN INTERSTITIAL CELL-DEFICIENT STRAIN. Journal of Cell Science. 1978;29: 17.

23. Holstein TW, Hobmayer E, David CN. Pattern of epithelial cell cycling in hydra. Developmental Biology. 1991;148: 602–611. doi:10.1016/0012-1606(91)90277-A

24. Chera S, Ghila L, Dobretz K, Wenger Y, Bauer C, Buzgariu W, et al. Apoptotic Cells Provide an Unexpected Source of Wnt3 Signaling to Drive Hydra Head Regeneration.

Developmental Cell. 2009;17: 279–289. doi:10.1016/j.devcel.2009.07.014
25. Bosch TCG, Anton-Erxleben F, Hemmrich G, Khalturin K. The Hydra polyp: Nothing but an active stem cell community: The Hydra polyp: a stem cell community. Development, Growth & Differentiation. 2009;52: 15–25. doi:10.1111/j.1440-169X.2009.01143.x
26. Passamaneck YJ, Martindale MQ. Cell proliferation is necessary for the regeneration of oral structures in the anthozoan cnidarian Nematostella vectensis. BMC Dev Biol. 2012;12: 34. doi:10.1186/1471-213X-12-34
27. DuBuc TQ, Traylor-Knowles N, Martindale MQ. Initiating a regenerative response; cellular and molecular features of wound healing in the cnidarian Nematostella vectensis. BMC Biol. 2014;12: 24. doi:10.1186/1741-7007-12-24
28. Amiel AR, Foucher K, Ferreira S, Röttinger E. Synergic coordination of stem cells is required to induce a regenerative response in anthozoan cnidarians. bioRxiv; 2019. p. 2019.12.31.891804. doi:10.1101/2019.12.31.891804
29. Schaffer AA, Bazarsky M, Levy K, Chalifa-Caspi V, Gat U. A transcriptional time-course analysis of oral vs. aboral whole-body regeneration in the Sea anemone Nematostella vectensis. BMC Genomics. 2016;17: 718. doi:10.1186/s12864-016-3027-1
30. McCusker C, Bryant SV, Gardiner DM. The axolotl limb blastema: cellular and molecular mechanisms driving blastema formation and limb regeneration in tetrapods: The Axolotl Limb Blastema. Regeneration. 2015;2: 54–71. doi:10.1002/reg2.32
31. Bryant SV, Endo T, Gardiner DM. Vertebrate limb regeneration and the origin of limb stem cells. The International Journal of Developmental Biology. 2002;46: 887–896.
32. Reddien PW, Alvarado AS. FUNDAMENTALS OF PLANARIAN REGENERATION. Annu Rev Cell Dev Biol. 2004;20: 725–757. doi:10.1146/annurev.cellbio.20.010403.095114
33. Aztekin C. Appendage regeneration is context dependent at the cellular level. Open Biol. 2021;11: 210126. doi:10.1098/rsob.210126
34. Bely AE, Nyberg KG. Evolution of animal regeneration: re-emergence of a field. Trends in Ecology & Evolution. 2010;25: 161–170. doi:10.1016/j.tree.2009.08.005
35. Leclère L, Röttinger E. Diversity of Cnidarian Muscles: Function, Anatomy, Development and Regeneration. Front Cell Dev Biol. 2017;4. doi:10.3389/fcell.2016.00157
