# Supplementary material for: Distinct stem-like cell populations facilitate functional regeneration of the Cladonema medusa tentacle
Source: PLoS Biol. 2023 Dec 21;21(12):e3002435. doi: 10.1371/journal.pbio.3002435 (PMC10734932; doi:10.1371/journal.pbio.3002435)
Supplement: Attachment — Submitted filename: Response to reviewer-revMS2-final2.pdf [file pbio.3002435.s014.pdf]

Response to reviewers' comments for ***PBIOLOGY-D-23-02278***

***Reviewer #1**: The authors have responded to my and the other reviewers' points from the first review cycle and revised the manuscript by performing new experiments, resulting in new or improved figures. They have also edited the text at places.*

***Comment 1.1**: The new data, in particular the 24 h EdU treatment that was followed by amputation and BrdU labeling, may indeed suggest that not all proliferative cells in the blastema directly derive from resident tentacle bulb i-cells. Therefore, it is possible that a distinct population (or populations) of cells also contributes to regeneration alongside i-cells and their progeny. This, however, isn't conclusive since the cell cycle length of Cladonema i-cells is yet unknown. A long i-cell cell cycle could still leave many i-cells unlabeled following a 24 h EdU treatment. These cells could increase their proliferation activity following amputation. Some of the other new experiments in the revised manuscript also make a better case for the authors' preferred conclusion; however, the work overall does not provide a definitive model re the cellular composition of the blastema.*

Assuming that there are indeed repair-specific cells in the blastema that are distinct from i-cells, the main question arising from this work remains their origin. The authors suggest two alternatives, one being a population of quiescent stem cells (would they call them i-cells too?), and the other is dedifferentiation of existing somatic cells.

Response 1.1:
We thank the reviewer for providing insightful comments and for acknowledging our efforts during revision. Our manuscript greatly improved by addressing their concerns. The reviewer still raised a concern about the potential long cell cycle of resident i-cells, which might lead to their contribution to blastema formation. Due to the limitation of extant techniques as well as available knowledge about *Cladonema*, at the current stage, we are not able to solve this issue. In response to the reviewer's comment as well as the editor's advice, we have added a section to the Discussion that includes limitations, alternative interpretations, and future directions. We have also revised the schematic of our tentacle regeneration model in Figure 7 (please see Figure 7A and 7B).

> (Revised manuscript; Page 26, Line 914-928)
>
> **Limitations, alternative interpretations, and future directions**
>
> Given the current lack of knowledge concerning the exact cell cycle and the inherent heterogeneity of *Cladonema* i-cells, it is plausible that a subset of i-cells located at

the bulb, not marked by 24 h EdU uptake, may become activated after amputation and contribute to blastema formation. Furthermore, the cellular origins of RSPCs in the blastema remain elusive, with at least two conceivable scenarios: mitotic re-entry of quiescent stem cells and/or dedifferentiation of neighboring specialized cells. Due to the current constraints imposed by available tools and techniques, addressing these issues is challenging at this stage.

Looking forward, it is essential to introduce genetic tools that allow the tracing of specific cell lineages and the manipulation of genes in *Cladonema*. This approach promises to deepen our understanding of jellyfish development and regeneration at the molecular and cellular levels and also to enhance the utility of *Cladonema* as a more useful research model.

Here we highlight revisions in Figures 7A and 7B.

Figure 7A (revision points were highlighted by purple circles for clarity)

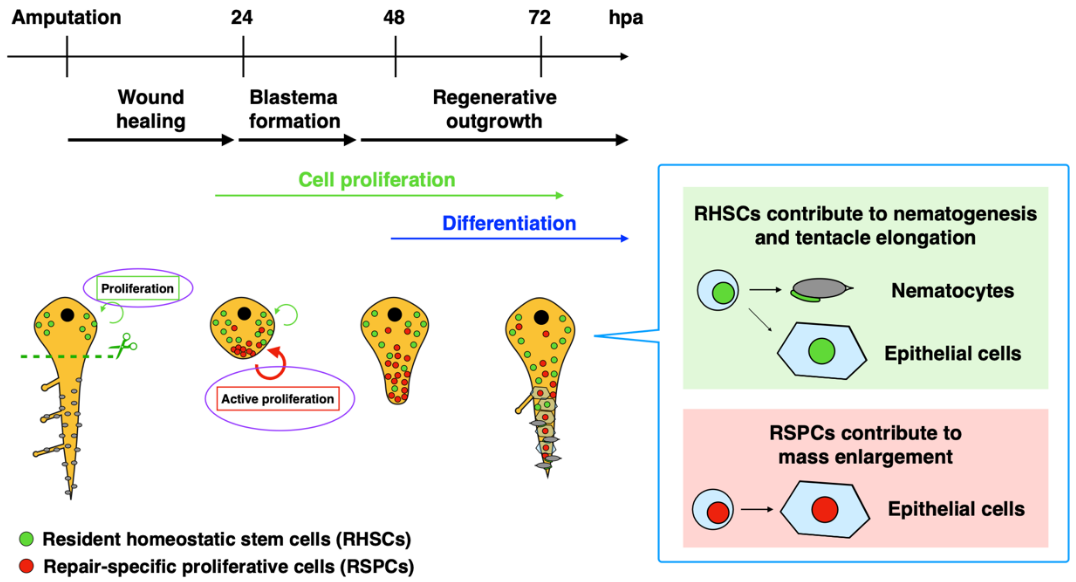

Figure 7B (revision points were highlighted by purple circles for clarity)

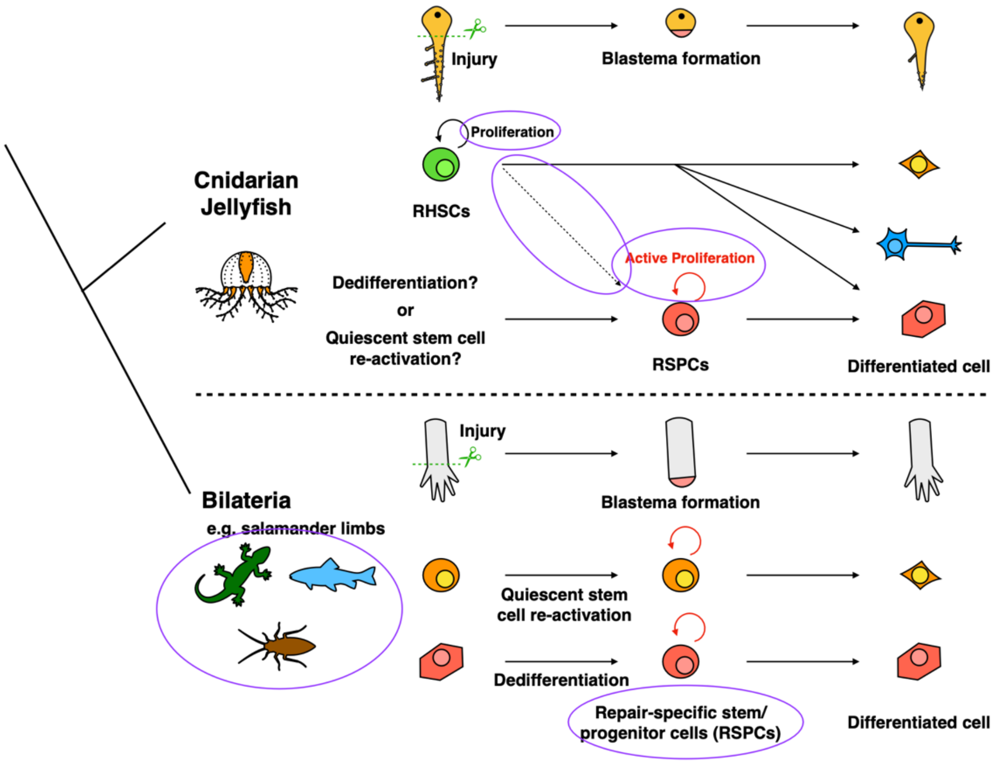

Minor comments:

***Comment 1.2**: The authors should avoid the term "lineage trace". This term refers to tracking single cells or at least a defined population that, e.g., expresses a marker gene (i.e., a lineage). What the authors performed was S-phase trace, looking at a broad group of cells that are in S-phase, which could encompass multiple lineages.*

Response 1.2:

As suggested, in the revised manuscript, we have used the term “nucleoside pulse-chase experiments” or “EdU and BrdU chase” when referring to chase experiments using nucleoside analogs, instead of “lineage trace.”

***Comment 1.3**: What is the evidence that cytoplasmic b-catenin marks all i-cells? Identifying i-cells by morphology may overestimate their numbers because they include committed i-cell progeny that still cycle (e.g., nematoblasts).*

Response 1.3:

Identification of cell types by the β-catenin antibody staining combines β-catenin localization and nuclear staining, which lead to detection of i-cells by the cytoplasmic

localization of β-catenin as well as by the presence of nucleoli. We chose this method because it has been established in different hydrozoans including *Hydractinia* as well as *Cladonema* [1–3].

However, as pointed by the reviewer, some early stages of nematoblasts are morphologically similar to i-cells as they are currently defined (Figure R1), and it is difficult to completely distinguish i-cells and nematoblasts with this technique. To avoid misunderstanding, we have revised the manuscript as follows:

(Revised manuscript; Page 16, Line 553-556)
"…we utilized the combination of β-catenin antibody staining and nuclear staining, which can distinguish i-cells (including i-cells and early nematoblasts), nematocytes, and epithelial cells (Fig 3A)."

Importantly, most nematoblasts do not actively proliferate based on the results that nematoblasts were not labeled by 1h EdU incubation (S8 Fig), and no RSPCs in blastema differentiate into nematocytes (Fig 6A-6C). These results suggest that nematoblasts around blastema are not included in RSPCs, and thus it does not impact the main conclusion of our work.

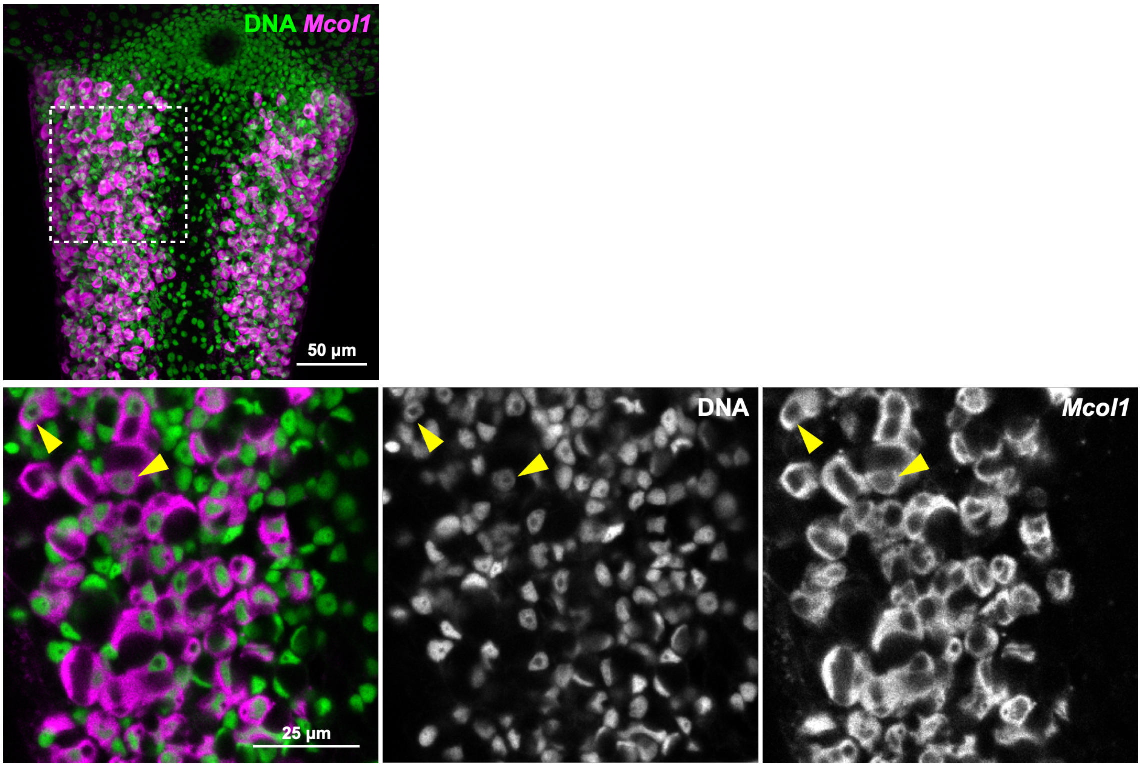

Figure R1. Early nematoblasts tend to exhibit prominent nucleoli. Nematoblasts in intact tentacle by *Minicollagen1(Mcol1)* FISH. Yellow arrowheads indicate early nematoblasts.

Comment 1.4*: Related to the above point, the authors should use the term i-cell consistently. First, they have to define them in Cladonema. As it stands now, it is not always clear to which cells the authors refer. Reading the manuscript, one gets the impression that the authors themselves aren't sure. Are i-cells merely small, proliferative cells with undifferentiated morphology, expressing Nanos/Piwi/Vasa, and being able to differentiate into some (which ones?) cell types? Alternatively, if they are unsure of how to define i-cells, they should clearly state it. This issue is always hard to resolve in a new animal model.*

Response 1.4:
Based on the definition of i-cells in other hydrozoan species including *Hydra* [4], *Hydractinia* [5], and *Clytia* [6,7], previous study [1] as well as ours defined the characteristics of *Cladonema* i-cells as follows: 1) morphological features like a large nucleus-to-cytoplasm ratio with prominent nucleoli, 2) the expression of stem cell marker genes such as *Nanos, Piwi,* and *Vasa,* 3) the ability to self-renew and differentiate into multiple cell types. For clarity, we have added this definition of i-cells at the beginning of the section of “Stem cells compose blastema” in Results.

> (Revised manuscript; Page 16, Line 547-553)
> Indeed, i-cells in hydrozoan jellyfish including *Clytia* and *Cladonema*, which are mainly localized at the tentacle bulb, are defined by 1) morphological features like a large nucleus-to-cytoplasm ratio with prominent nucleoli, 2) the expression of stem cell marker genes such as *Nanos1* and *Piwi*, and 3) the ability to self-renew and differentiate into multiple cell types [39,46,47]. It is thus possible that undifferentiated stem cells like i-cells contribute to blastema.

References

1. Hou S, Zhu J, Shibata S, Nakamoto A, Kumano G. Repetitive accumulation of interstitial cells generates the branched structure of *Cladonema* medusa tentacles. Development. 2021;148: dev199544. doi:10.1242/dev.199544

2. Plickert G, Frank U, Müller WA. Hydractinia, a pioneering model for stem cell biology and reprogramming somatic cells to pluripotency. Int J Dev Biol. 2012;56: 519–534. doi:10.1387/ijdb.123502gp

3. Frank U, Plickert G, Müller WA. Cnidarian Interstitial Cells: The Dawn of Stem Cell Research. In: Rinkevich B, Matranga V, editors. Stem Cells in Marine Organisms. Dordrecht: Springer Netherlands; 2009. pp. 33–59. doi:10.1007/978-90-481-2767-2_3

4. Holstein TW. The Hydra stem cell system – Revisited. Cells & Development. 2023;174: 203846. doi:10.1016/j.cdev.2023.203846

5. Gahan JM, Bradshaw B, Flici H, Frank U. The interstitial stem cells in Hydractinia and their role in regeneration. Current Opinion in Genetics & Development. 2016;40: 65–73. doi:10.1016/j.gde.2016.06.006

6. Leclère L, Jager M, Barreau C, Chang P, Le Guyader H, Manuel M, et al. Maternally localized germ plasm mRNAs and germ cell/stem cell formation in the cnidarian Clytia. Developmental Biology. 2012;364: 236–248. doi:10.1016/j.ydbio.2012.01.018

7. Chari T, Weissbourd B, Gehring J, Ferraioli A, Leclère L, Herl M, et al. Whole-animal multiplexed single-cell RNA-seq reveals transcriptional shifts across *Clytia* medusa cell types. Sci Adv. 2021;7: eabh1683. doi:10.1126/sciadv.abh1683
